# The effect of sodium restricted diet on the prognosis of heart failure patients: a systemic review and meta-analysis

**DOI:** 10.3389/fcvm.2026.1751581

**Published:** 2026-05-01

**Authors:** Ming-hao Ma, Zhi-cheng Yang, Yuan-yao Cao, Yuan Cao, Zhi-hao Wang

**Affiliations:** 1 The Second Qilu Hospital, Cheeloo College of Medicine, Shandong University, Jinan, Shandong, China; 2 School of Nursing and Rehabilitation, Shandong University, Jinan, Shandong, China; 3 National Key Laboratory for Innovation and Transformation of Luobing Theory, The Key Laboratory of Cardiovascular Remodeling and Function Research, Chinese Ministry of Education, Chinese National Health Commission and Chinese Academy of Medical Sciences, Department of Cardiology, Qilu Hospital of Shandong University, Jinan, Shandong, China; 4 Department of Geriatric Medicine & Laboratory of Gerontology and Anti-Aging Research, Qilu Hospital of Shandong University, Jinan, Shandong, China

**Keywords:** heart failure, meta-analysis, mortality, prognosis, readmission, sodium restricted diet

## Abstract

**Aims:**

To evaluate whether a sodium restricted diet can improve the prognosis of HF patients.

**Methods:**

Retrieve for randomized controlled trials (RCTs) on sodium restricted diets for HF patients from PubMed, Web of Science, Cochrane Library and Embase, with a retrieval period from the establishment of databases to July 2025. Two researchers independently conducted literature screening using RevMan 5.4 and evaluated the quality using Cochrane risk of bias tool. Finally, StataMP 18 was used for meta-analysis.

**Results:**

16 RCTs were included, with 2260 patients. The meta-analysis results showed that compared with the control group, the sodium restricted diet group had significantly increased all-cause mortality rate (RR 1.50 [95%CI: 1.05–2.14] I^2^=0) and cardiac mortality rate (RR 2.51 [95%CI: 1.55–4.05] I^2^=7.5%), while there was no statistically significant difference in HF readmission rate and all-cause readmission rate between the two groups (*p* > 0.05). Subgroup analyses showed that in the subgroup of HFrEF patients and the subgroup that simultaneously restricted fluid intake and used diuretics, the sodium restricted diet was associated with an increased risk of all-cause death and HF readmission. Descriptive analyses showed that a sodium restricted diet might improve patients' quality of life, but had no significant effect on serum NT-proBNP level.

**Conclusion:**

Although sodium restriction may improve the quality of life of HF patients, it is not associated with a reduced risk of readmission or lower the serum NT-proBNP level; for HFrEF patients receiving diuretic therapy and fluid restriction, the sodium restricted diet is associated with an increased risk of all-cause mortality and HF readmission.

## Graphical Abstract

**Figure:**
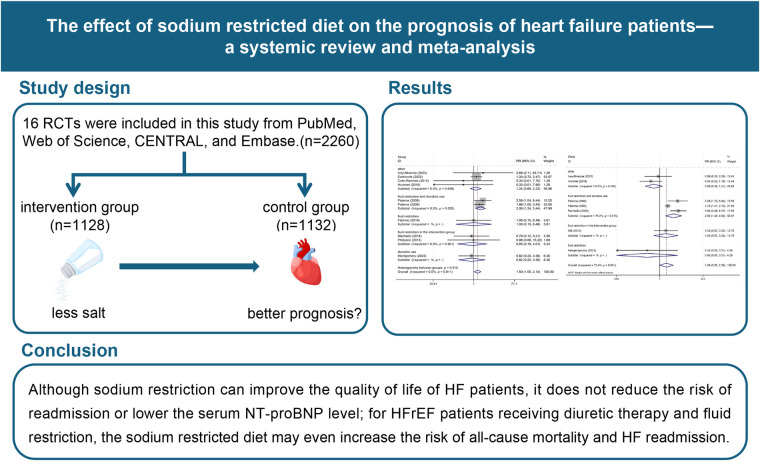

## 1 Introduction

Heart failure is a clinical syndrome caused by abnormal cardiac structure and function, characterized by decreased pumping function and inability to meet the metabolic needs. Its occurrence is correlated with the progression of cardiovascular diseases such as myocardial infarction and hypertension (1). In recent years, with the lasting development of treatment for heart failure and its primary diseases, the survival rate and quality of life of HF patients have been improved (2). However, as the aging population continues to increase, the current status of heart failure remains severe, imposing a heavy burden on the social economy and medical resources. According to Report on Cardiovascular Health and Diseases in China 2024, a total of 14.29 million HF patients were admitted to China in 2023, with an average age of (71.2 ± 12.6) years old and a 30-day readmission rate of 11.0% (3). According to *HF STATS 2024*, approximately 6.7 million Americans over the age of 20 suffer from heart failure, while the lifetime risk of heart failure is up to 24% (4).

Sodium restriction, as one of the lifestyle interventions, is recommended for comprehensive management of heart failure. *Chinese Guidelines for the Diagnosis and Treatment of Heart Failure 2024* and *2021 ESC Guidelines for the diagnosis and treatment of acute and chronic heart failure* respectively recommend that HF patients control their daily sodium intake within 3 gram and 2 gram (5, 6). In the development of heart failure, due to insufficient renal blood flow perfusion, the renin-angiotensin-aldosterone system (RAAS) is activated, which not only produces pressor effect by contracting vascular smooth muscle, but also promotes reabsorption of kidney, further aggravating fluid retention (7). Moderate sodium restriction can improve water and sodium retention and reduce blood volume, thereby reducing cardiac preload, and alleviating dyspnea, chest tightness, edema and other symptoms. However, in recent years, systemic reviews and meta-analyses on the impact of sodium restricted diets in heart failure have lacked analysis of heart failure biomarkers. This article is aimed to provide evidence-based support for clinical practice through the systemic review and meta-analysis of randomized controlled trials on sodium restricted diets in patients with heart failure.

## 2 Methods

### 2.1 Literature retrieval strategy

Retrieve randomized controlled trials on sodium restricted diet for HF patients from PubMed, Web of Science, Cochrane Library, and Embase using a combination of subject words and free words. The terms used are “Heart Failure/Cardiac Failure/Heart Decompensation/Congestive Heart Failure/Right Sided Heart Failure/Left Sided Heart Failure/Myocardial Failure”, “Sodium/Salt”, and “low/restrict*/reduc*”. The retrieval period is from the establishment of the database to July 2025. The detailed search strategies are available in the Supplementary materials.

### 2.2 Inclusion and exclusion criteria

Inclusion criteria: (1) The research type is randomized controlled trial; (2) The research subjects are adult patients (≥ 18 years old) diagnosed with heart failure; (3) The intervention is sodium restriction (≤ 3 gram sodium per day), and the sodium intake of the control group is higher than that of the intervention group; (4) Report at least one outcome measure: NT-proBNP level, quality of life (QoL) score, all-cause readmission, HF readmission, all-cause mortality, or cardiogenic mortality.

Exclusion criteria: (1) Inability to obtain full-text or duplicate publications; (2) The type of the article is review, commentary, letter, conference abstract, etc; (3) The quality assessment result is low; (4) Data is missing or cannot be converted; (5) Non-adult populations (< 18 years old); (6) Studies in which the intervention was hypertonic saline infusion or other non-dietary sodium restriction measures without sodium restriction; (7) Patients with severe renal dysfunction or other severe comorbidities (e.g., advanced liver disease, active malignancy) that may affect sodium metabolism or outcomes.

### 2.3 Literature screening and data extraction

Retrieve relevant literature and import it into EndNote X9 for management. Two researchers conduct literature screening by independently reading the title, abstract, and full-text of articles based on established inclusion and exclusion criteria. After completing the screening separately, the two researchers cross-check the results. In case of disagreement, two researchers will reach a consensus through discussion; If there is still a dispute after discussion, the third researcher will make an independent judgment and make the final decision. Subsequently, relevant information is extracted and recorded using Excel.

### 2.4 Literature quality assessment

Using the Cochrane risk of bias tool to evaluate the quality of screened randomized controlled trials, bias assessment mainly included seven aspects: random sequence generation, allocation concealment, blinding of participants and personnel, blinding of outcome assessment, incomplete outcome data, selective reporting, and other bias. The risk level is divided into low risk, unclear or high risk. Exclude studies with a quality rating of C (one or more aspects are evaluated as high risk).

### 2.5 Statistical methods

Meta-analysis is conducted using StataMP 18. For binary variables, use relative risk (RR) and its 95% confidence interval (95%CI) as the combined effect size. For continuous variables, if the data is presented in the form of mean and standard deviation (Mean ± SD), use mean deviation (MD) and its 95%CI as the combined effect size; if the data is presented in the form of median and quartile (median [IQR]), descriptive analysis should be used. Assess heterogeneity by I^2^ and Q statistic, *P*≤0.1 or I^2^≥50% indicates significant heterogeneity between studies, and random effects model should be used for analysis; *P* > 0.1 or I2 < 50% indicates low heterogeneity between studies, and fixed effects model should be used for analysis. Subgroup analyses are conducted based on LVEF (HFpEF, HFrEF, or HFpEF and HFrEF), NYHA class (≤ IV, ≤ III or ≤ II), mean/median age (≥ 70 years old or < 70 years old), sodium restriction level (2–3 gram sodium per day, 1–2 gram sodium per day or < 1 gram sodium per day), intervention period (≤ 1 month, > 1 month and ≤ 3 months or > 3 months), follow-up period (≤ 1 month, > 1 month and ≤ 3 months or > 3 months) and co-intervention measures (fluid restriction and diuretic use, fluid restriction, diuretic use, or none) to evaluate the impact of these factors on the meta-analysis results. Sensitivity analysis, along with subgroup analyses, is used to analyze heterogeneity sources and evaluate the robustness of the results. Egger's test and Begg's test are used to assess publication bias. *P* < 0.05 in Egger's test or Begg's test indicates significant publication bias.

## 3 Results

### 3.1 Literature screening results

**Figure 1:**
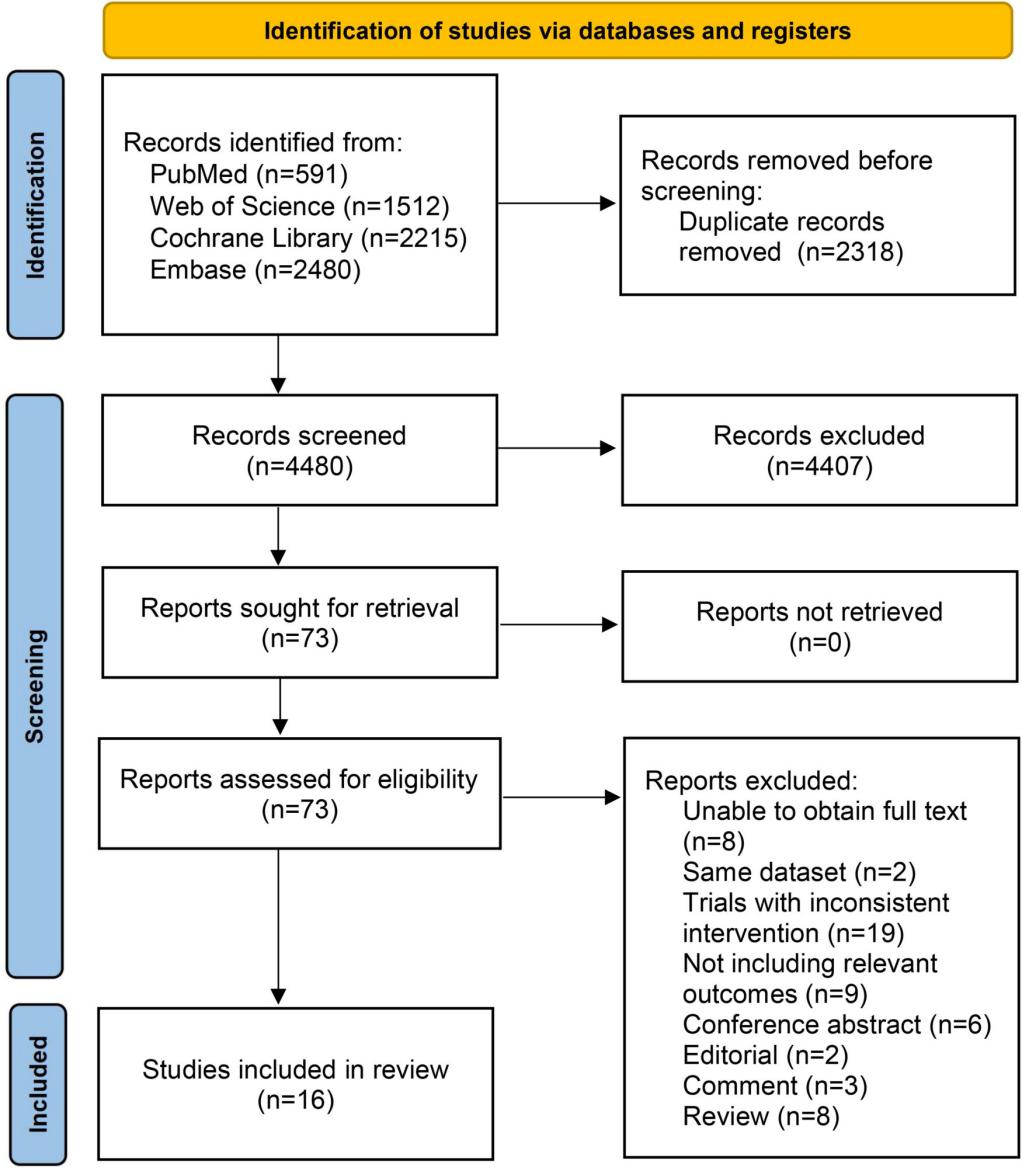
Literature screening process.

6798 articles were included in the preliminary retrieval, and after excluding duplicate articles, 4480 articles remained. After initial screening and re-screening, 16 articles were finally included (8–23). The detailed literature screening process is shown in Figure 1.

### 3.2 Characteristics of included articles

A total of 2260 patients were included in the studies, including 1128 patients in the intervention group who received a clear sodium restricted diet, and 1132 patients in the control group who had a higher daily sodium intake than the intervention group. The characteristics and intervention details of the included articles are shown in Tables 1, 2.

**Table 1:** Characteristics of included articles.

First Author	Country	Year	Types of heart failure	NYHA class	Renal function	Age (years old)	Sample Size (n)
						Sodium restriction group/Control group	Sodium restriction group/Control group
Aliti	Brazil	2013	Acute decompensated HFrEF	II/III/IV	Estimated creatinine clearance > 30mL/min/1.73 m2	60.6 ± 10.5/59.3 ± 12.2	38/37
Colin-Ramirez	Canada	2004	HFrEF or HFpEF	I/II/III	Patients with renal failure were excluded	64.2 ± 16.2/59.9 ± 16.4	30/35
Colin-Ramirez	Canada	2015	Chronic HFrEF or HFpEF	II/III	eGFR > 20mL/min/1.73 m2	66.1 (58.0–71.4)/63.9 (51.6–76.9)	19/19
Ezekowitz	Australia, etc	2022	Chronic HFrEF or HFpEF	I/II/III/IV	eGFR > 20mL/min/1.73 m2	66 (57–73)/67 (58–75)	397/409
Fabricio	Brazil	2019	Acute decompensated HFrEF or HFpEF	-	Estimated creatinine clearance > 30mL/min/1.73 m2	59.5 ± 11.9/56.4 ± 12.3	22/22
Hummel	USA	2018	Acute decompensated HFrEF or HFpEF	-	Estimated creatinine clearance > 30mL/min/1.73 m2	71 ± 8/70 ± 8	33/33
Ivey-Miranda	Mexico	2023	Chronic HFrEF	I/II	Estimated creatinine clearance > 30mL/min/1.73 m2	61 ± 12/58 ± 13	37/33
Kalogeropoulos	USA	2019	HFrEF	-	Patients with renal replacement therapy or Stage 4 or 5 CKD were excluded	59.5 ± 9.1/63.9 ± 11.4	12/15
Machado d'Almeida	Brazil	2018	Decompensated HFpEF	II/III/IV	eGFR > 30mL/min/1.73 m2	73.7 ± 11.1/70.4 ± 12.6	30/23
Montgomery	USA	2023	Acute HFrEF or HFpEF	-	eGFR > 15mL/min/1.73 m2	70 ± 13/70 ± 12	31/34
Nakasato	Brazil	2010	Compensated HFrEF	I/II/III	Creatinine < 2.5mg/dL	52 ± 2/52 ± 2	25/25
Parrinello	Italy	2009	Compensated congestive HFrEF	II	Creatinine < 2mg/dL, BUN < 60mg/dL	72.5 ± 8/73.1 ± 7	87/86
Paterna	Italy	2008	Compensated congestive HFrEF	II	Creatinine < 2mg/dL, BUN < 60 mg/dL	73.3 ± 9/72.1 ± 7	114/118
Paterna	Italy	2009	Compensated HFrEF	II	Creatinine < 2mg/dL, BUN < 60mg/dL	-	205/205
Philipson	Sweden	2010	Chronic HFrEF or HFpEF	II/III	Patients with renal dysfunction (creatinine > 250 µmol/L) were excluded	74 ± 8/74 ± 9	17/13
Philipson	Sweden	2013	Chronic HFrEF or HFpEF	II/III	Patients with renal dysfunction (creatinine > 250 µmol/L) were excluded	74 ± 8.6/76 ± 7.5	49/48

Age is shown as mean ± standard deviation, or median (quartile 1-quartile 3). HFrEF: heart failure with reduced ejection fraction; HFpEF: heart failure with preserved ejection fraction; NYHA, New York Heart Association; eGFR, estimated glomerular filtration rate, BUN, blood urea nitrogen; CKD, chronic kidney disease.

**Table 2:** Intervention details of included articles.

First Author	Country	Year	Intervention/Follow-up Period	Sodium intake of the intervention group	Sodium intake of the control group	Other Interventions
Aliti	Brazil	2013	7 days/1 month	0.8 gram sodium per day	3–5 gram sodium per day	The intervention group’s fluid intake was restricted to 800 mL per day
Colin-Ramirez	Canada	2004	6 months	2–2.4 gram sodium per day	Follow diet suggestions	The intervention group’s fluid intake was restricted to 1.5 L per day
Colin-Ramirez	Canada	2015	6 months	1.5 gram sodium per day	2.3 gram sodium per day	-
Ezekowitz	Australia, etc	2022	12 months/12 months	1.5 gram sodium per day	Follow clinical suggestions	-
Fabricio	Brazil	2019	7 days	1.2 gram sodium per day	2.8 gram sodium per day	Fluid intake was restricted to 1 L per day
Hummel	USA	2018	12 weeks	1.5 gram sodium per day	Follow standardized education	-
Ivey-Miranda	Mexico	2023	20 weeks	2 gram sodium per day	3 gram sodium per day	-
Kalogeropoulos	USA	2019	12 weeks/12 weeks	1.5 gram sodium per day	3 gram sodium per day	Fluid intake was restricted to 2 L per day
Machado d’Almeida	Brazil	2018	7 days/1 month	0.8 gram sodium per day	4 gram sodium per day	The intervention group’s fluid intake was restricted to 800 mL per day
Montgomery	USA	2023	4 days/3 months	0.8 gram sodium per day	0.8 gram sodium per day + 0.8 gram sodium/2 capsules, tid	Intravenous injection of furosemide > 10 mg per hour
Nakasato	Brazil	2010	7 days	0.8 gram sodium per day	2.4 gram sodium per day	Fluid intake was restricted to 1 L per day
Parrinello	Italy	2009	6 months	1.8 gram sodium per day	2.8 gram sodium per day	Oral administration of 250–500 mg furosemide, bid, fluid intake was restricted to 1 L per day
Paterna	Italy	2008	6 months	1.8 gram sodium per day	2.8 gram sodium per day	Oral administration of 250–500 mg furosemide, bid, fluid intake was restricted to 1 L per day
Paterna	Italy	2009	6 months	1.8 gram sodium per day	2.8 gram sodium per day	Oral administration of 250–500 mg furosemide, bid, fluid intake was restricted to 1–2 L per day
Philipson	Sweden	2010	12 weeks	2–3 gram sodium per day	Follow ESC guidelines	The intervention group’s fluid intake was restricted to 1.5 L per day
Philipson	Sweden	2013	12 weeks/12 months	2–3 gram sodium per day	Follow clinical suggestions	The intervention group’s fluid intake was restricted to 1.5 L per day

ESC, european society of cardiology.

### 3.3 Literature quality assessment

**Figure 2:**
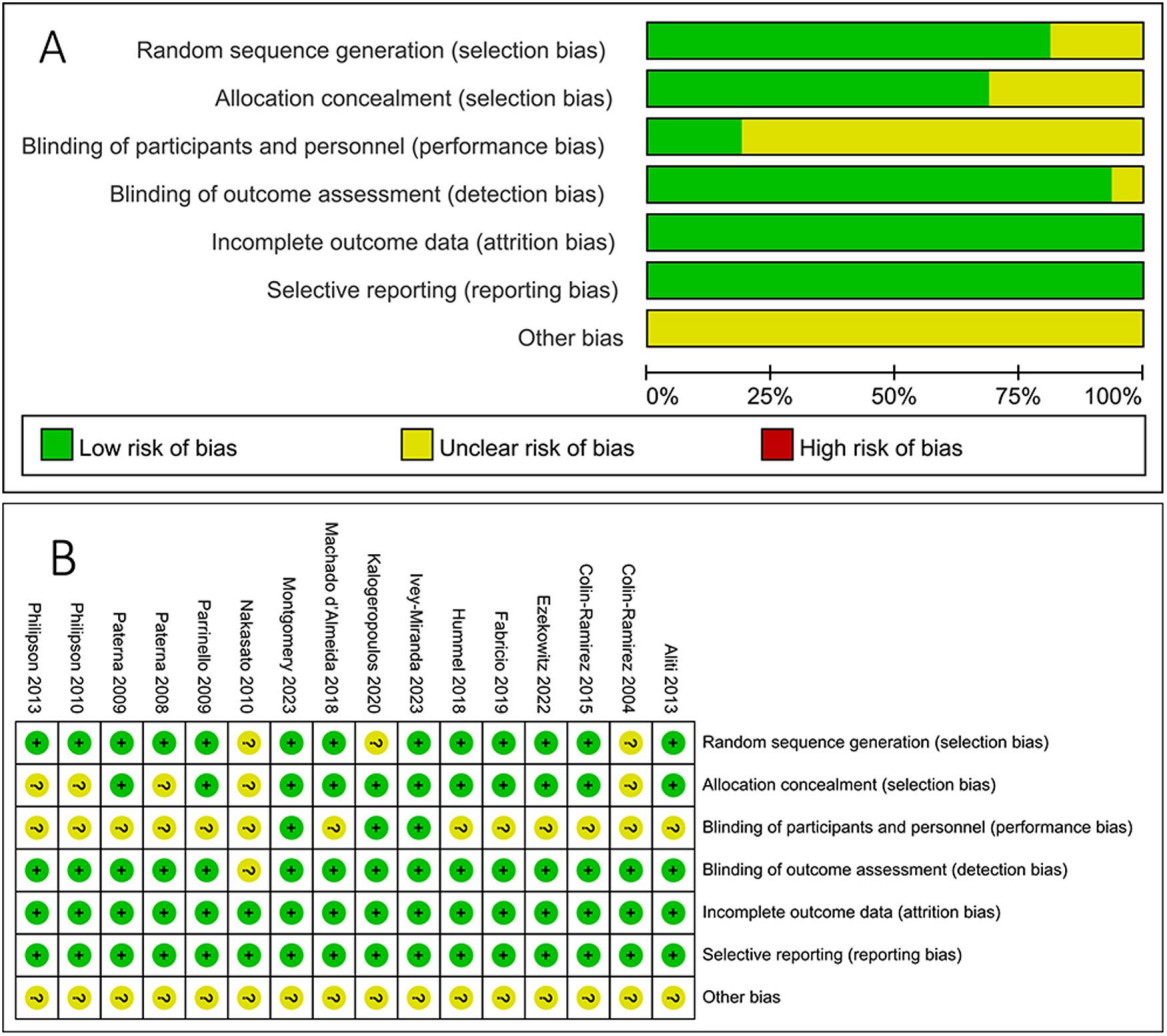
Results of risk of bias assessment **(A)** risk of bias graph; **(B)** risk of bias summary.

All included studies are randomized controlled trials. 13 articles described specific randomization methods and were evaluated as low-risk; 11 articles clearly stated that randomization was conducted by specialized personnel independent of the researchers and were evaluated as low-risk; due to the limitations of intervention measures, only 3 articles were double-blind trials and were evaluated as low-risk; 15 articles explicitly implemented blinding for outcome assessors and were evaluated as low-risk. All 16 articles were rated as B level. No article was excluded for high risk in one or more aspects in the quality assessment. As shown in Figure 2.

### 3.4 Meta-analysis results

#### 3.4.1 All-cause mortality

**Figure 3:**
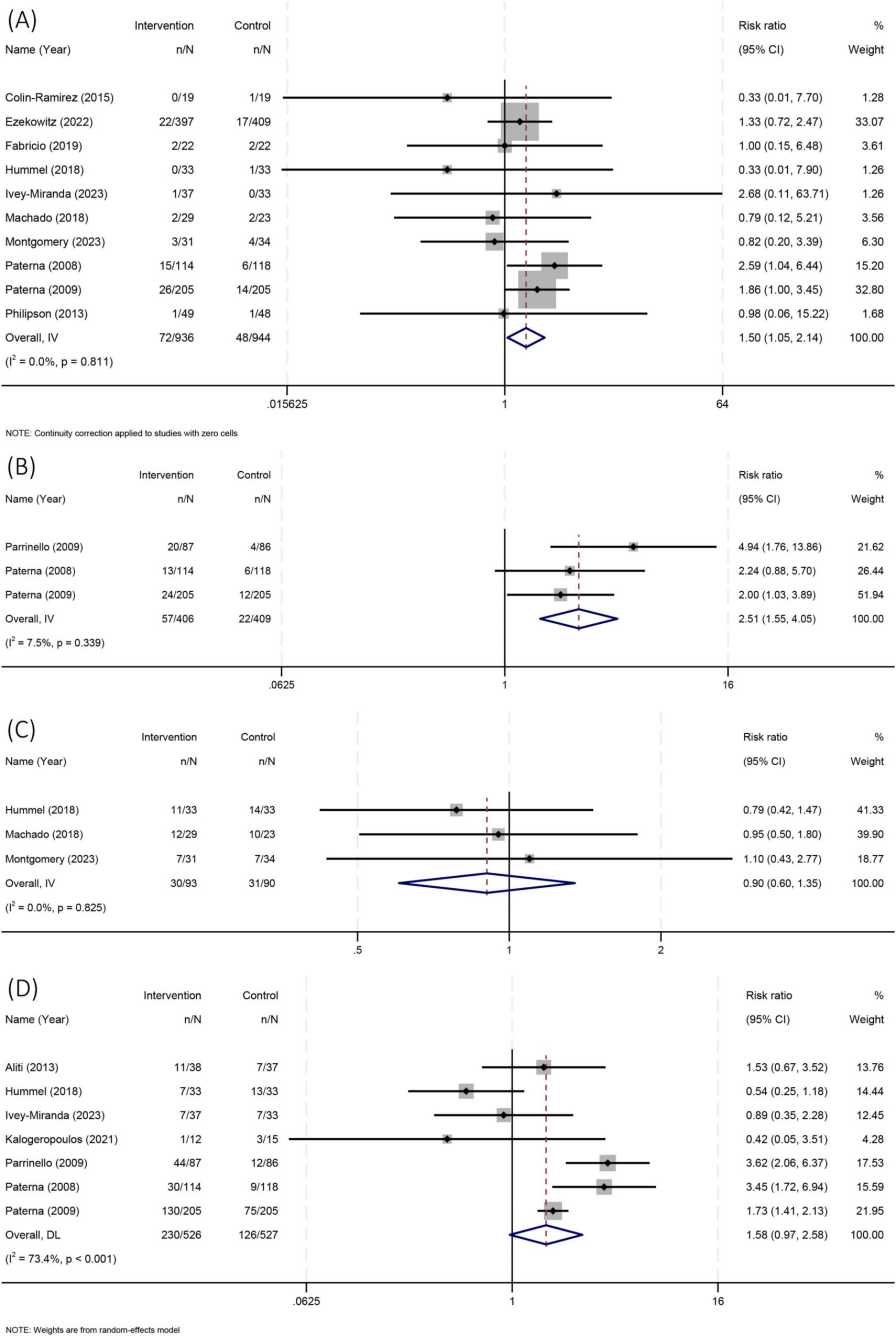
Forest plot of all-cause mortality. **(A)** All-cause mortality; **(B)** Cardiogenic mortality; **(C)** All-cause readmission; **(D)** HF readmission.

Ten articles reported all-cause mortality rate, involving a total of 1880 patients (10–14, 16, 17, 20, 21, 23). There were 72 all-cause deaths (7.69%) in the sodium restriction group and 48 all-cause deaths (5.08%) in the control group. There is no heterogeneity among the studies (I^2^=0, *P*=0.811), and fixed effects model was used for analysis. The results showed that a sodium restricted diet was associated with an increased risk of all-cause death for HF patients (RR 1.50 [95% CI: 1.05–2.14]). As shown in Figure 3A.

#### 3.4.2 Cardiogenic mortality

Three articles reported cardiogenic mortality rate, involving a total of 815 patients (19–21). There were 57 cardiogenic deaths (14.03%) in the sodium restriction group and 22 cardiogenic deaths (5.38%) in the control group. There is low heterogeneity among the studies (I^2^=7.5%, *P*=0.339), and fixed effects model was used for analysis. The results showed that a sodium restricted diet was associated with an increased risk of cardiogenic death for HF patients (RR 2.51 [95% CI: 1.55–4.05]). As shown in Figure 3B.

#### 3.4.3 All-cause readmission

Three articles reported all-cause readmission rate, involving a total of 183 patients (13, 16, 17). 30 patients (32.26%) were readmitted to hospital in the sodium restriction group, while 31 patients (34.44%) were readmitted to hospital in the control group. There is no heterogeneity among the studies (I^2^=0, *P*=0.825), and fixed effects model was used for analysis. The results showed that a sodium restricted diet had no significant effect on all-cause readmission for HF patients (RR 0.90 [95%CI: 0.60–1.35]). As shown in Figure 3C.

#### 3.4.4 HF readmission

Seven articles reported HF readmission rate, involving a total of 1053 patients (8, 13–15, 19–21). 230 patients (43.73%) were readmitted to hospital in the sodium restriction group, while 126 patients (23.91%) were readmitted to hospital in the control group. There is high heterogeneity among the studies (I^2^=73.4%, *P* < 0.001), and random effects model was used for analysis. The results showed that a sodium restricted diet had no significant effect on HF readmission for HF patients (RR 1.58 [95%CI: 0.97–2.58]). As shown in Figure 3D.

#### 3.4.5 Subgroup analyses results of all-cause mortality

##### 3.4.5.1 Stratified by LVEF

(1) For the subgroup of HFrEF or HFpEF which included six studies, there was no significant difference in all-cause mortality between the sodium restriction group and the control group (RR 1.12 [95%CI: 0.67–1.88]) (2). For the HFrEF subgroup which included three studies, the all-cause mortality rate in the sodium restriction group was higher than that in the control group (RR 2.08 [95%CI: 1.25–3.44]) (3). For the HFpEF subgroup which included one study, there was no significant difference in all-cause mortality between the sodium restriction group and the control group (RR 0.79 [95%CI: 0.12–5.21]). As shown in Supplementary Figure 1.

##### 3.4.5.2 Stratified by NYHA class

(1) For the NYHA ≤ II subgroup which included three studies, the all-cause mortality rate in the sodium restriction group was higher than that in the control group (RR 2.08 [95%CI: 1.25–3.44]) (2) For the NYHA ≤ III subgroup which included two studies, there was no significant difference in all-cause mortality between the sodium restriction group and the control group (RR 0.61 [95%CI: 0.08–4.85]) (3). For the NYHA ≤ IV subgroup which included two studies, there was no significant difference in all-cause mortality between the sodium restriction group and the control group (RR 1.27 [95%CI: 0.70–2.28]) (4). For the subgroup in which NYHA class was not reported in the included three studies, there was no significant difference in all-cause mortality between the two groups (RR 0.79 [95%CI: 0.27–2.29]). As shown in Supplementary Figure 2.

##### 3.4.5.3 Stratified by mean/median age

(1) For the ≥70 years old subgroup which included six studies, the all-cause mortality rate in the sodium restriction group was higher than that in the control group (RR 1.67 [95%CI: 1.06–2.64]) (2). For the <70 years old subgroup which included four studies, there was no significant difference in all-cause mortality between the sodium restriction group and the control group (RR 1.27 [95%CI: 0.72–2.24]). As shown in Supplementary Figure 3.

##### 3.4.5.4 Stratified by sodium restriction level

(1) For the 2–3 gram sodium per day subgroup which included two studies, there was no significant difference in all-cause mortality between the sodium restriction group and the control group (RR 1.51 [95%CI: 0.19–12.00]) (2). For the 1–2 gram sodium per day subgroup which included six studies, the all-cause mortality rate in the sodium restriction group was higher than that in the control group (RR 1.61 [95%CI: 1.10–2.35]) (3). For the <1 gram sodium per day subgroup which included two studies, there was no significant difference in all-cause mortality between the sodium restriction group and the control group (RR 0.81 [95%CI: 0.26–2.52]). As shown in Supplementary Figure 4.

##### 3.4.5.5 Stratified by intervention period

(1) For the >3 months subgroup which included five studies, the all-cause mortality rate in the sodium restriction group was higher than that in the control group (RR 1.69 [95%CI: 1.15–2.50]) (2). For the >1 month and ≤3 months subgroup which included two studies, there was no significant difference in all-cause mortality between the sodium restriction group and the control group (RR 0.62 [95%CI: 0.08–4.90]) (3). For the ≤1 month subgroup which included three studies, there was no significant difference in all-cause mortality between the sodium restriction group and the control group (RR 0.86 [95%CI: 0.33–2.26]). As shown in Supplementary Figure 5.

##### 3.4.5.6 Stratified by follow-up period

(1) For the >3 months subgroup which included six studies, the all-cause mortality rate in the sodium restriction group was higher than that in the control group (RR 1.68 [95%CI: 1.14–2.46]) (2). For the >1 month and ≤3 months subgroup which included two studies, there was no significant difference in all-cause mortality between the sodium restriction group and the control group (RR 0.71 [95%CI: 0.19–2.58]) (3). For the ≤1 month subgroup which included two studies, there was no significant difference in all-cause mortality between the sodium restriction group and the control group (RR 0.89 [95%CI: 0.24–3.36]). As shown in Supplementary Figure 6.

##### 3.4.5.7 Stratified by co-intervention measures

(1) For the subgroup of fluid restriction and diuretics use which included two studies, the all-cause mortality rate in the sodium restriction group was higher than that in the control group (RR 2.06 [95%CI: 1.24–3.44]) (2). For the subgroup of fluid restriction which included three studies, there was no significant difference in all-cause mortality between the sodium restriction group and the control group (RR 0.91 [95%CI: 0.27–2.99]) (3). For the subgroup of diuretics use which included one study, there was no significant difference in all-cause mortality between the sodium restriction group and the control group (RR 0.82 [95%CI: 0.20–3.39]) (4). For the subgroup with no co-intervention which included four studies, there was no significant difference in all-cause mortality between the sodium restriction group and the control group (RR 1.24 [95%CI: 0.69–2.23]). As shown in Supplementary Figure 7.

#### 3.4.6 Subgroup analyses results of HF readmission

##### 3.4.6.1 Stratified by LVEF

(1) For the HFrEF or HFpEF subgroup which included one study, there was no significant difference in HF readmission rate between the sodium restriction group and the control group (RR 0.54 [95%CI: 0.25–1.18]) (2). For the HFrEF subgroup which included six studies, the HF readmission rate in the sodium restriction group was higher than that in the control group (RR 1.95 [95%CI: 1.25–3.03]). As shown in Supplementary Figure 8.

##### 3.4.6.2 Stratified by NYHA class

(1) For the NYHA ≤ II subgroup which included four studies, the HF readmission rate in the sodium restriction group was higher than that in the control group (RR 2.18 [95%CI: 1.29–3.70]) (2). For the NYHA ≤ IV subgroup which included one study, there was no significant difference in HF readmission rate between the sodium restriction group and the control group (RR 1.53 [95%CI: 0.67–3.52]) (3). For the subgroup in which NYHA class was not reported in the included two studies, there was no significant difference in HF readmission rate between the sodium restriction group and the control group (RR 0.52 [95%CI: 0.25–1.09]). As shown in Supplementary Figure 9.

##### 3.4.6.3 Stratified by mean/median age

(1) For the ≥70 years old subgroup which included four studies, there was no significant difference in HF readmission rate between the sodium restriction group and the control group (RR 1.90 [95%CI: 1.00–3.64]) (2). For the <70 years old subgroup which included three studies, there was no significant difference in HF readmission rate between the sodium restriction group and the control group (RR 1.11 [95%CI: 0.61–2.02]). As shown in Supplementary Figure 10.

##### 3.4.6.4 Stratified by sodium restriction level

(1) For the 2–3 gram sodium per day subgroup which included one study, there was no significant difference in HF readmission rate between the sodium restriction group and the control group (RR 0.89 [95%CI: 0.35–2.28]) (2). For the 1–2 gram sodium per day subgroup which included five studies, there was no significant difference in HF readmission rate between the sodium restriction group and the control group (RR 1.72 [95%CI: 0.91–3.23]) (3). For the <1 gram sodium per day subgroup which included one study, there was no significant difference in HF readmission rate between the sodium restriction group and the control group (RR 1.53 [95%CI: 0.67–3.52]). As shown in Supplementary Figure 11.

##### 3.4.6.5 Stratified by intervention period

(1) For the >3 months subgroup which included four studies, the HF readmission rate in the sodium restriction group was higher than that in the control group (RR 2.18 [95%CI: 1.29–3.70]) (2). For the >1 month and ≤3 months subgroup which included two studies, there was no significant difference in HF readmission rate between the sodium restriction group and the control group (RR 0.52 [95%CI: 0.25–1.09]) (3). For the ≤1 month subgroup which included one study, there was no significant difference in HF readmission rate between the sodium restriction group and the control group (RR 1.53 [95%CI: 0.67–3.52]). As shown in Supplementary Figure 12.

##### 3.4.6.6 Stratified by follow-up period

(1) For the >3 months subgroup which included five studies, the HF readmission rate in the sodium restriction group was higher than that in the control group (RR 2.00 [95%CI: 1.18–3.39]) (2). For the >1 month and ≤3 months subgroup which included one study, there was no significant difference in HF readmission rate between the sodium restriction group and the control group (RR 0.54 [95%CI: 0.25–1.18]) (3). For the ≤1 month subgroup which included one study, there was no significant difference in HF readmission rate between the sodium restriction group and the control group (RR 1.53 [95%CI: 0.67–3.52]). As shown in Supplementary Figure 13.

##### 3.4.6.7 Stratified by co-intervention measures

(1) For the subgroup of fluid restriction and diuretics use which included three studies, the HF readmission rate in the sodium restriction group was higher than that in the control group (RR 2.62 [95%CI: 1.49–4.62]) (2). For the subgroup of fluid restriction which included two studies, there was no significant difference in HF readmission rate between the sodium restriction group and the control group (RR 1.17 [95%CI: 0.42–3.28]) (3). For the subgroup with no co-intervention which included two studies, there was no significant difference in HF readmission rate between the sodium restriction group and the control group (RR 0.66 [95%CI: 0.36–1.21]). As shown in Supplementary Figure 14.

#### 3.4.6 Sensitivity analysis and heterogeneity analysis

**Figure 4:**
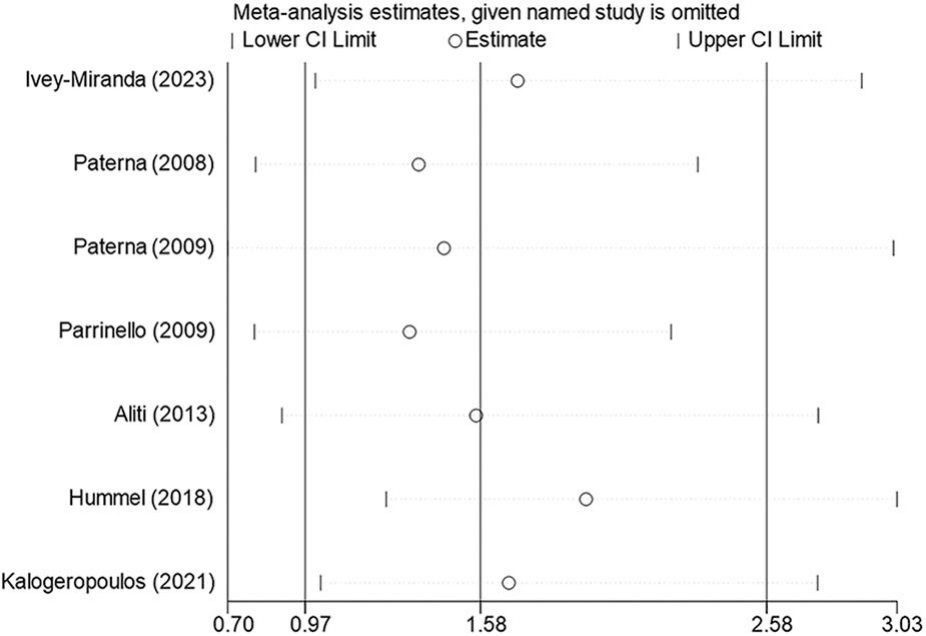
Sensitivity analysis of HF readmission.

High heterogeneity was found in HF readmission rate when conducting the meta-analysis on mortality and readmission. Sensitivity analysis was conducted by excluding studies sequentially. The combined effect size of HF readmission after excluding any research was not statistically significant and consistent with the original combined effect size. As shown in Figure 4.

However, in the subgroup analyses of HF readmission, we observed that high intra-subgroup heterogeneity existed in the subgroup with both fluid restriction and diuretic use when stratified by co-intervention measures (I^2^=76.2%, *P*=0.015), which was derived from Supplementary Figure 14. This subgroup included three studies (Parrinello 2009, Paterna 2008, Paterna 2009) (19–21), and the combined effect size showed that sodium restriction combined with fluid restriction and diuretic use was associated with an increased risk of HF readmission (RR 2.62 [95%CI: 1.49–4.62]). Further exploration of potential sources of this intra-subgroup heterogeneity revealed that it might be attributed to differences in the specific dosage of diuretics administered, the strictness of fluid restriction, and the baseline characteristics of included patients.

Notably, the inter-subgroup heterogeneity test for co-intervention measures yielded a statistically significant result (*P*=0.005), indicating that the effect of sodium restriction on HF readmission varied significantly across different co-intervention subgroups. Compared to the “fluid restriction and diuretic use” subgroup, the “fluid restriction” subgroup (RR 1.17 [95%CI: 0.42–3.28]) and “no co-intervention” subgroup (RR 0.66 [95%CI: 0.36–1.21]) showed no statistically significant associations. In contrast to the high intra-subgroup heterogeneity in the “fluid restriction and diuretic use” subgroup, other co-intervention subgroups had low or no intra-subgroup heterogeneity (fluid restriction: I^2^=19.4%, *P*=0.265; no co-intervention: I^2^=0.0%, *P*=0.418). This contrast suggests that the combination of fluid restriction and diuretic use not only modifies the effect of sodium restriction on HF readmission but also introduces greater variability in study results within the subgroup.

#### 3.4.7 Publication bias

Publication bias analysis was conducted on all-cause mortality. The Egger's test result showed *P*=0.289, while the Begg's test result showed *P*=0.721, indicating no publication bias.

### 3.5 Quality of life

Nine articles reported quality of life (9–11, 13–15, 18, 22, 23), three studies used generic scales for evaluation (9, 22, 23); while six studies used disease-specific scales (10, 11, 13–15, 18), including two studies using the Minnesota Living with Heart Failure Questionnaire (14, 18) and four studies using the Kansas City Cardiomyopathy Questionnaire (10, 11, 13, 15). Six articles reported an improvement in quality of life of HF patients in both the sodium restriction group and the control group (9–11, 13, 14, 18). Among them, five articles showed that the sodium restriction group had a higher degree of improvement in quality of life than the control group (9–11, 13, 18); one study showed that there was a nearly significant change (*P*=0.052) in quality of life in the control group compared to the sodium restriction group (14). Two articles showed that only the control group had a certain degree of improvement in quality of life (22, 23). One article showed that only the sodium restriction group had an improvement in quality of life (*P* < 0.001) (15). Overall, a sodium restricted diet may improve quality of life of HF patients.

### 3.6 Serum NT-proBNP level

Six articles reported serum NT-proBNP level (12, 14, 15, 17, 22, 23). Two articles showed a decrease in NT-proBNP level in both groups (12, 17), three articles showed no significant change in NT-proBNP level in both groups (14, 15, 22), and one study showed an increase in NT-proBNP level in both groups (23). The difference between groups was not statistically significant in all six articles (12, 14, 15, 17, 22, 23). Overall, a sodium restricted diet has no significant effect on serum NT-proBNP level of HF patients.

## 4 Discussion

This study showed that a sodium restricted diet was associated with an increased risk of all-cause mortality and cardiogenic mortality and might improve quality of life of HF patients, but had no significant effect on all-cause readmission rate, HF readmission rate, and serum NT-proBNP level in HF patients. Subgroup analysis revealed that these adverse effects were particularly evident among patients with reduced ejection fraction, those of advanced age, individuals with milder heart failure symptoms, and during prolonged intervention periods. Most notably, the combination of sodium restriction with concurrent fluid restriction and diuretic therapy emerged as the strongest predictor of adverse outcomes, suggesting this specific co-intervention pattern represents the primary driver of the observed mortality and readmission risks, rather than dietary sodium restriction alone.

The results of this study were consistent with the meta-analysis published by Colin-Ramirez et al. in terms of readmission and quality of life (24); however, in terms of all-cause mortality, the results were inconsistent and might be related to the inclusion of studies that restricted fluid intake and used diuretics in both groups. For HFrEF patients with evidence of fluid retention, diuretics are commonly recommended (5). Loop and thiazide diuretics inhibit sodium reabsorption (25); when combined with dietary sodium restriction, this readily lowers serum sodium levels, elevates aldosterone and plasma renin activity, and worsens renal parameters, as directly observed in the included studies (19–21). It is easy to induce hyponatremia with lowered blood sodium level by restricting sodium intake simultaneously. Research has shown that hyponatremia increases the risk of readmission and mortality in HF patients, and is associated with poor prognosis in HF patients (26). Severe hyponatremia not only causes electrolyte imbalance, but also promotes the movement of water into cells, leading to cerebral edema and neurological symptoms, even threatening the patient's life (27). Low blood sodium level can also activate RAAS and the sympathetic nervous system, leading to vasoconstriction and increased myocardial contractility, thereby exacerbating cardiac function worsening (7). A meta-analysis based on 10 studies published by Diaz-Arocutipa et al. showed that on the basis of a sodium restricted diet, the use of hypertonic saline in combination with furosemide can reduce the mortality and readmission rate of patients with acute decompensated heart failure (ADHF) (28). In addition, this study found that a sodium restricted diet had no significant effect on NT-proBNP level in HF patients. NT-proBNP is a heart failure biomarker produced in ventricular myocytes by enzymatic cleavage of BNP precursor and released into the bloodstream when ventricular volume loading and ventricular pressure increase (29). The sodium restricted diet mainly reduces the sodium intake to reduce water and sodium retention, thus reducing the circulating blood volume and reducing the cardiac preload. Early HF patients can still maintain normal cardiac function through the Frank Starling mechanism and neurohumoral effects, but with prolonged compensatory time, the cytotoxic effects of neurohumoral regulation promote myocardial fibrosis and ventricular remodeling, ultimately leading to a sustained decrease in overall myocardial contractility and compliance (30). Even if sodium restriction reduces blood volume, ventricular pressure may still remain at a high level, making it difficult to effectively reduce myocardial wall stress. The decrease in blood volume may also trigger compensatory regulation in the body, by releasing angiotensin II, aldosterone, and norepinephrine to increase vascular resistance and stimulate NT-proBNP secretion.

Current clinical guidelines often recommend that HF patients adopt a sodium restricted diet to control blood volume and reduce cardiac burden. However, this study shows that a sodium restricted diet is associated with an increased risk of readmission and mortality in HFrEF patients who also receive diuretics therapy and fluid restriction, suggesting the need to re-evaluate this recommendation. Specifically, for HFrEF patients on diuretics and fluid restriction, we recommend maintaining sodium intake at 2.0–3.0 gram per day rather than aggressive restriction (<2.0 gram per day); for those without these concurrent interventions, moderate restriction to 1.5–2.0 gram per day may be acceptable with monitoring; elderly patients (≥70 years) should avoid strict restriction and maintain ≥2.0 g per day; it may be appropriate for HFpEF patients with a restriction to 2.0–3.0 gram per day individualized to comorbidities; for acute HF patients, restricting sodium intake below 1gram per day is not recommended.

The guidelines for heart failure should be based on evidence-based research, highlighting personalized dietary guidance, and providing more accurate sodium intake recommendations for patients with different NYHA class, comorbidities (such as renal insufficiency), and different treatment plans. When providing health education to HF patients, medical systems and community health service institutions should not simply emphasize the importance of sodium restriction, but should inform patients of the potential risks associated with such a diet, especially for HFrEF patients who receive diuretics therapy and fluid restriction. In addition, a comprehensive follow-up management system for HF patients should be established to monitor patients' dietary compliance, cardiac function indicators to avoid hyponatremia caused by sodium restriction.

This study has following limitations: (1) The sample sizes of the included studies are generally small, meaning that a few large-sample studies exert a significant impact on the meta-analysis results (8, 18, 19). (2) Some articles did not introduce specific randomization methods or allocation concealment; (3) Included articles vary in regions, sodium restriction levels and other intervention measures, and differences in dietary habits and heart failure treatment plan between different countries have an impact on the applicability of the study results; (4) Some outcome measures showed high heterogeneity, suggesting potential variability across studies in patient characteristics, intervention protocols, or follow-up period; (5) Many included studies concurrently applied fluid restriction and diuretic therapy alongside sodium restriction, which introduce co-intervention effects and limit the ability to distinguish the independent contribution of sodium restriction from the combined treatment regimen; (6) The limited number of researches included in some outcome measures and subgroups has an impact on the reliability of the study results; (7) Although some subgroup analyses did not show statistical significance, the insufficient sample size in these subgroups may limit the statistical power to detect true differences, and the non-significant results should be interpreted with caution.

## Data Availability

The original contributions presented in the study are included in the article/Supplementary Material, further inquiries can be directed to the corresponding author.
