# Supplementary figures and images for: The effect of sodium restricted diet on the prognosis of heart failure patients: a systemic review and meta-analysis
Source: Front Cardiovasc Med. 2026 May 1;13:1751581. doi: 10.3389/fcvm.2026.1751581 (PMC13176190; doi:10.3389/fcvm.2026.1751581)

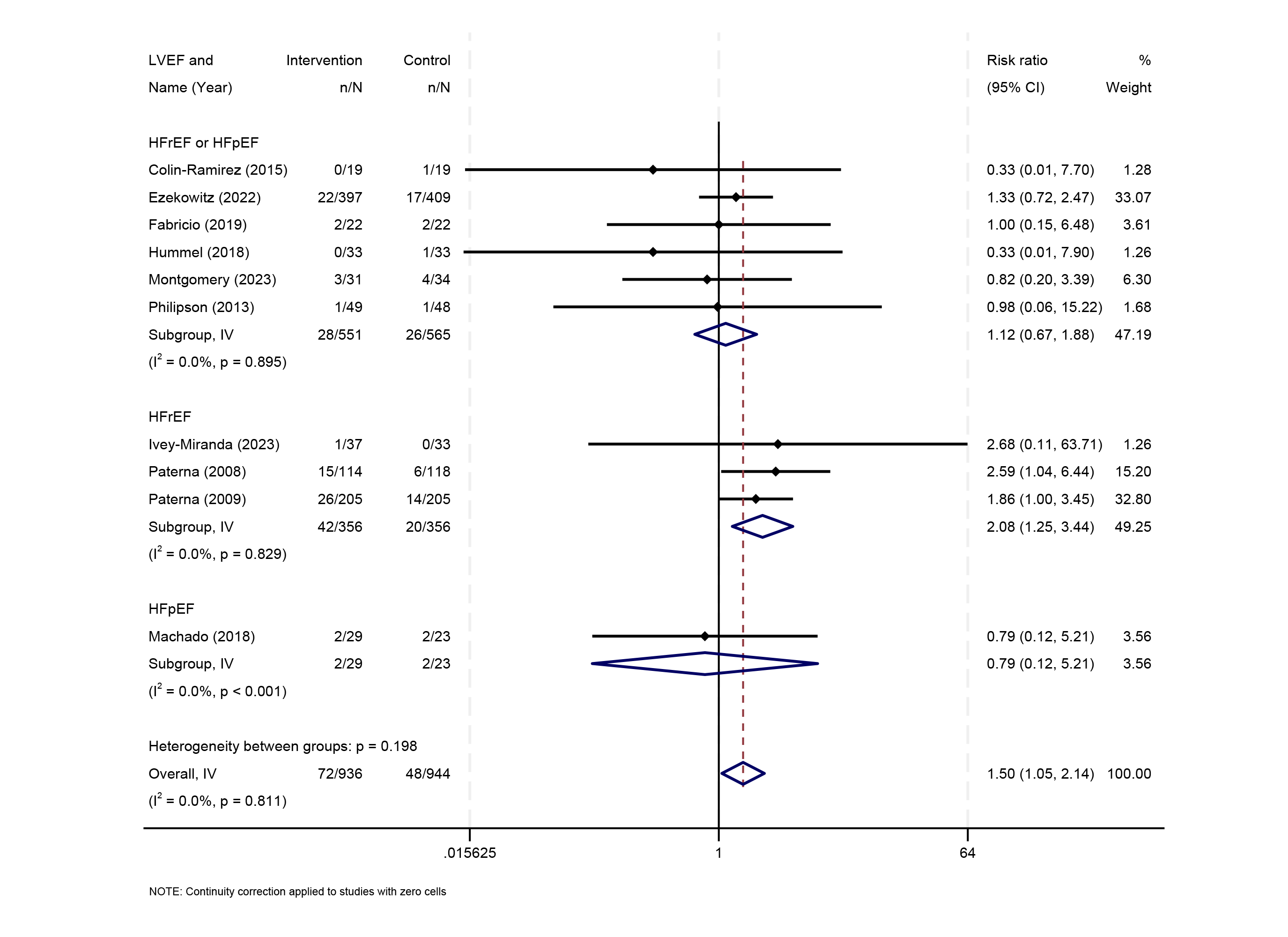

Supplement: Supplementary file 1 [file Image1.tif]

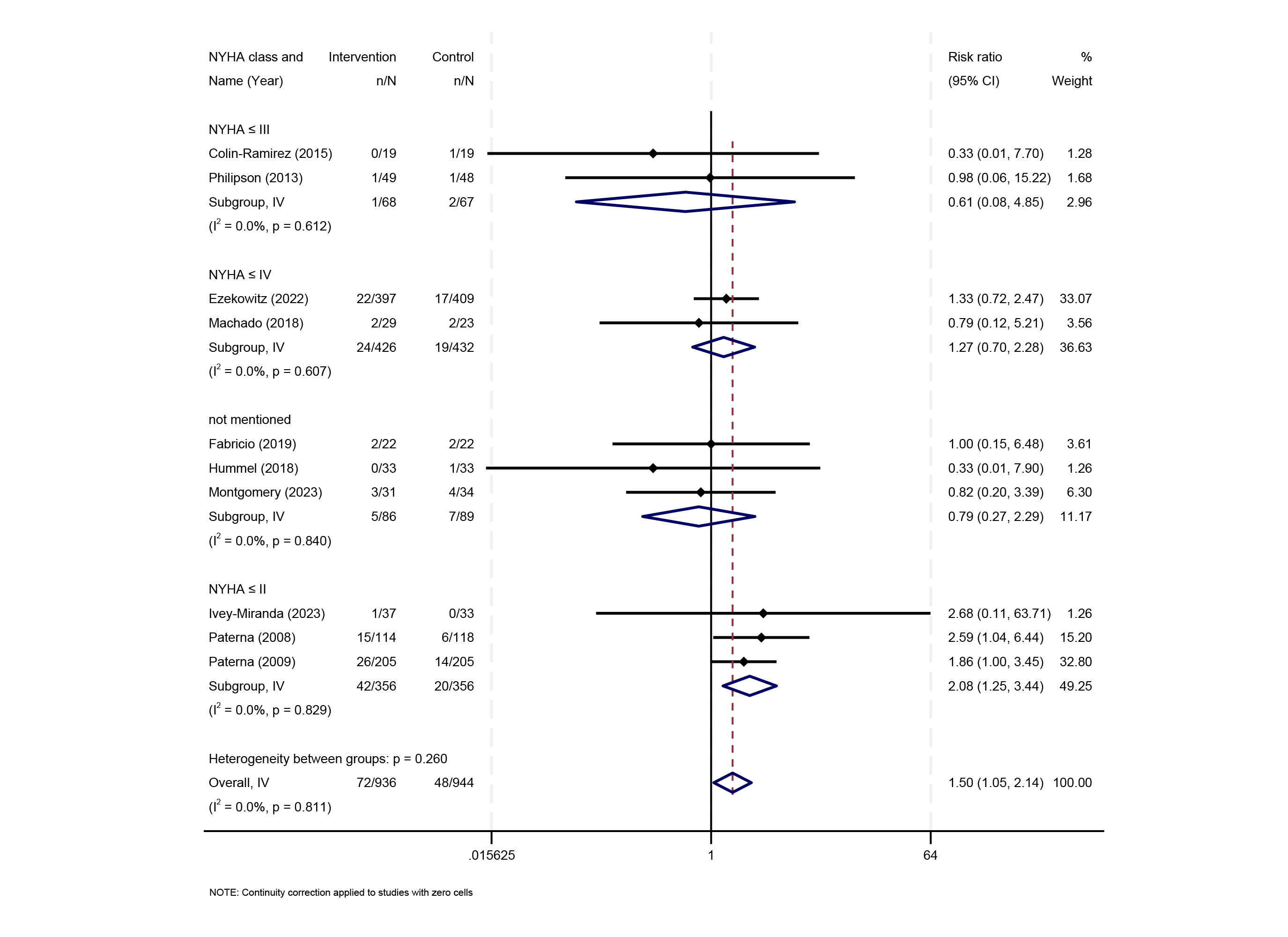

Supplement: Supplementary file 2 [file Image2.tif]

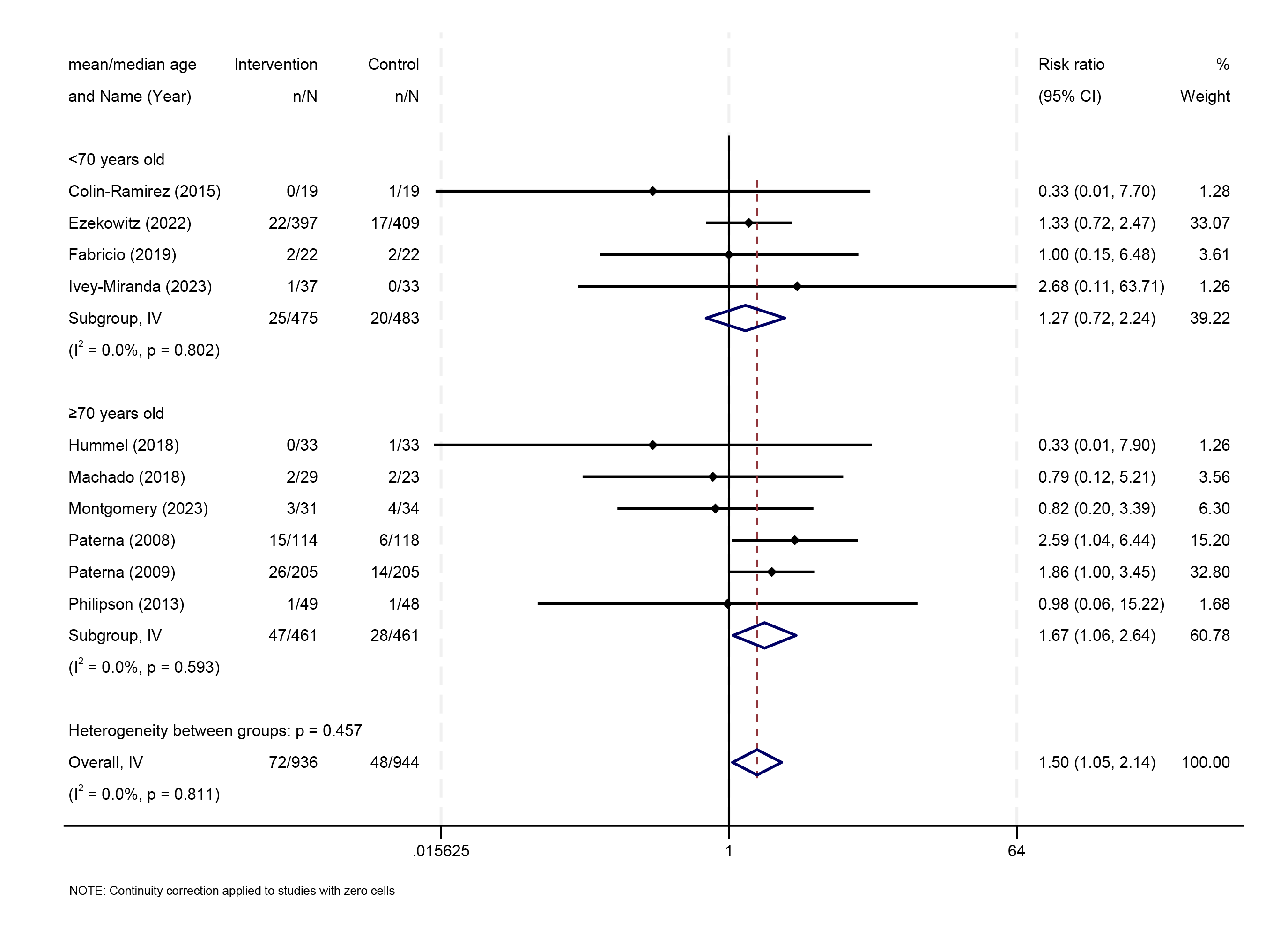

Supplement: Supplementary file 3 [file Image3.tif]

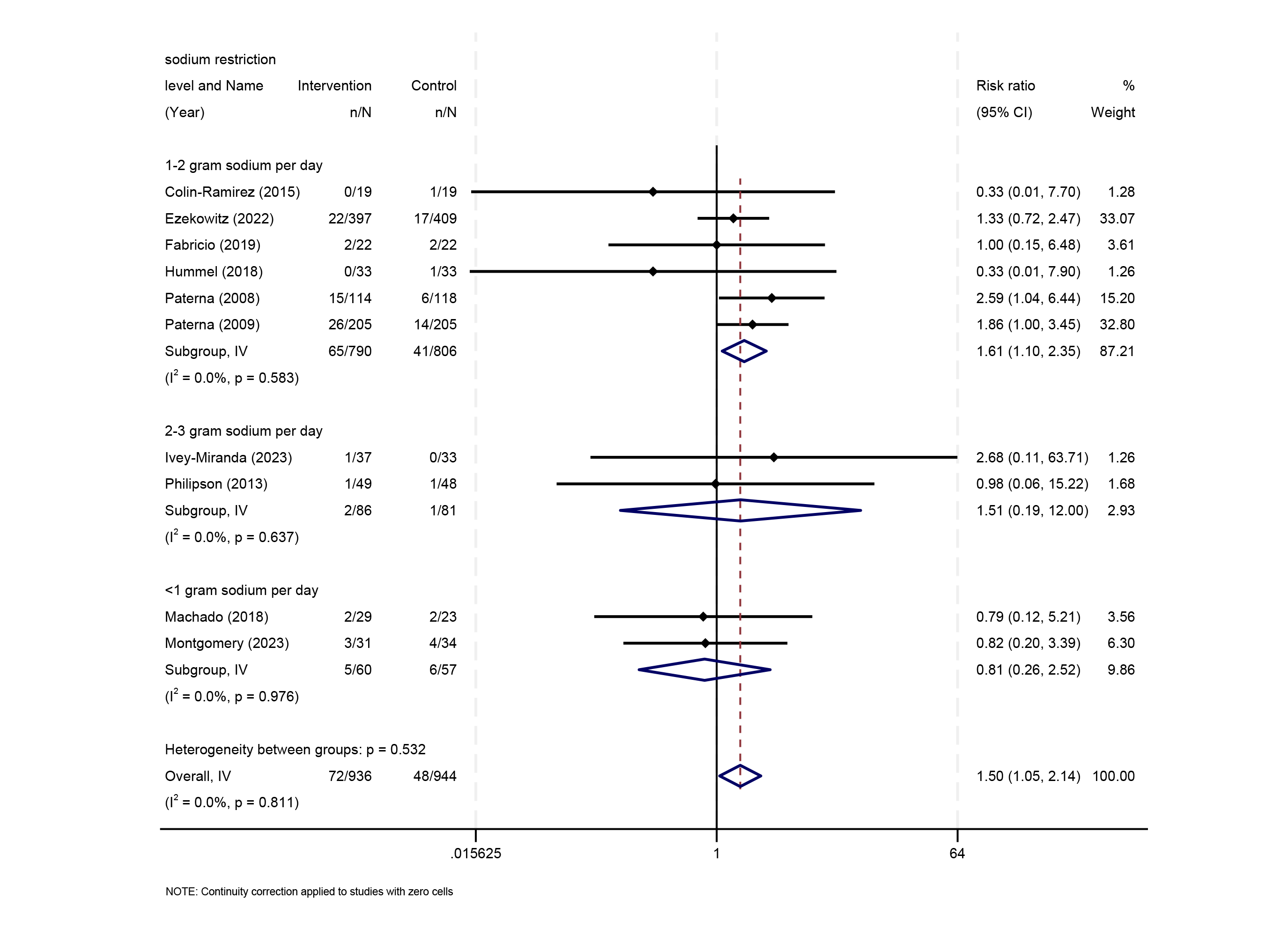

Supplement: Supplementary file 4 [file Image4.tif]

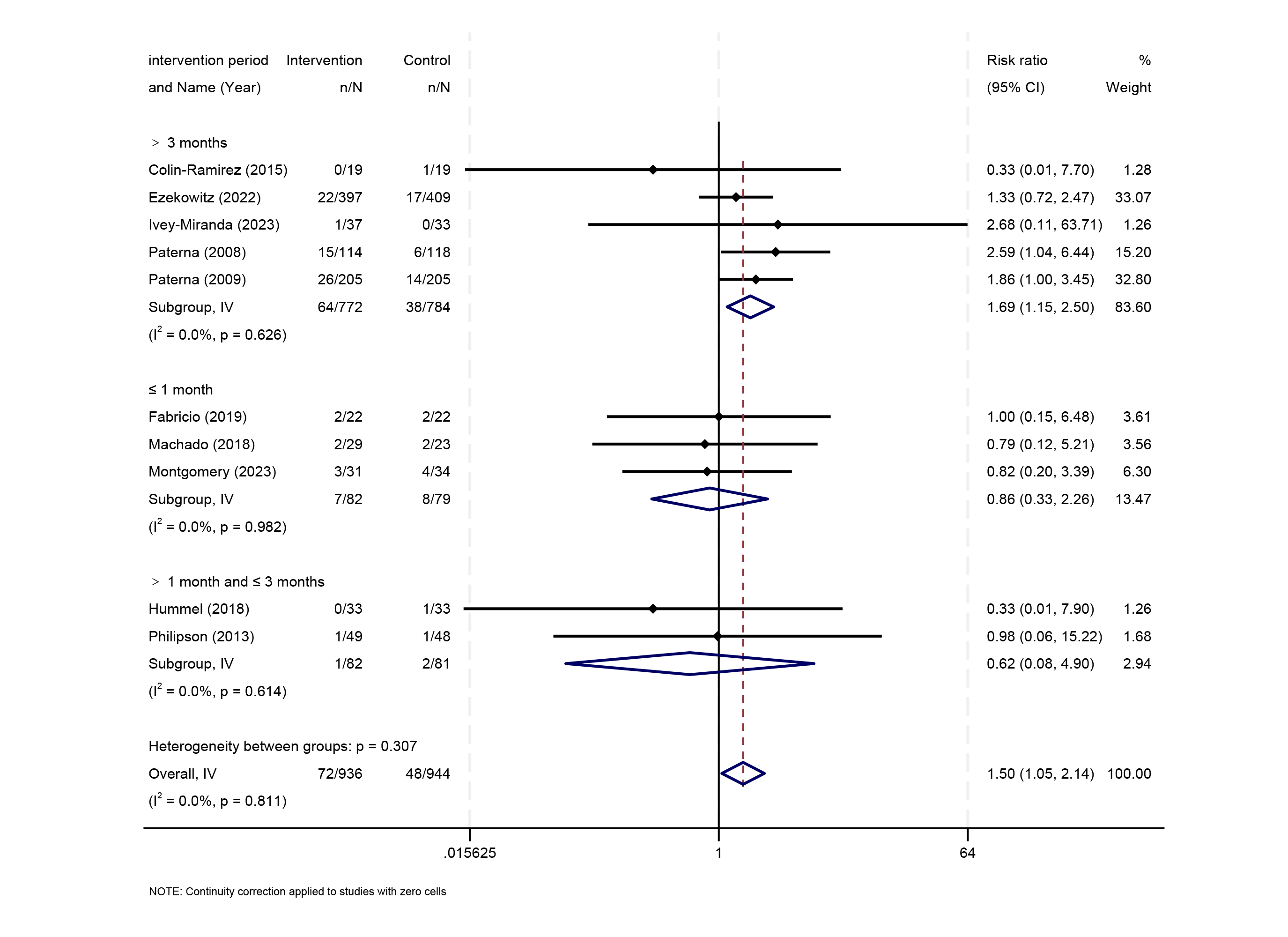

Supplement: Supplementary file 5 [file Image5.tif]

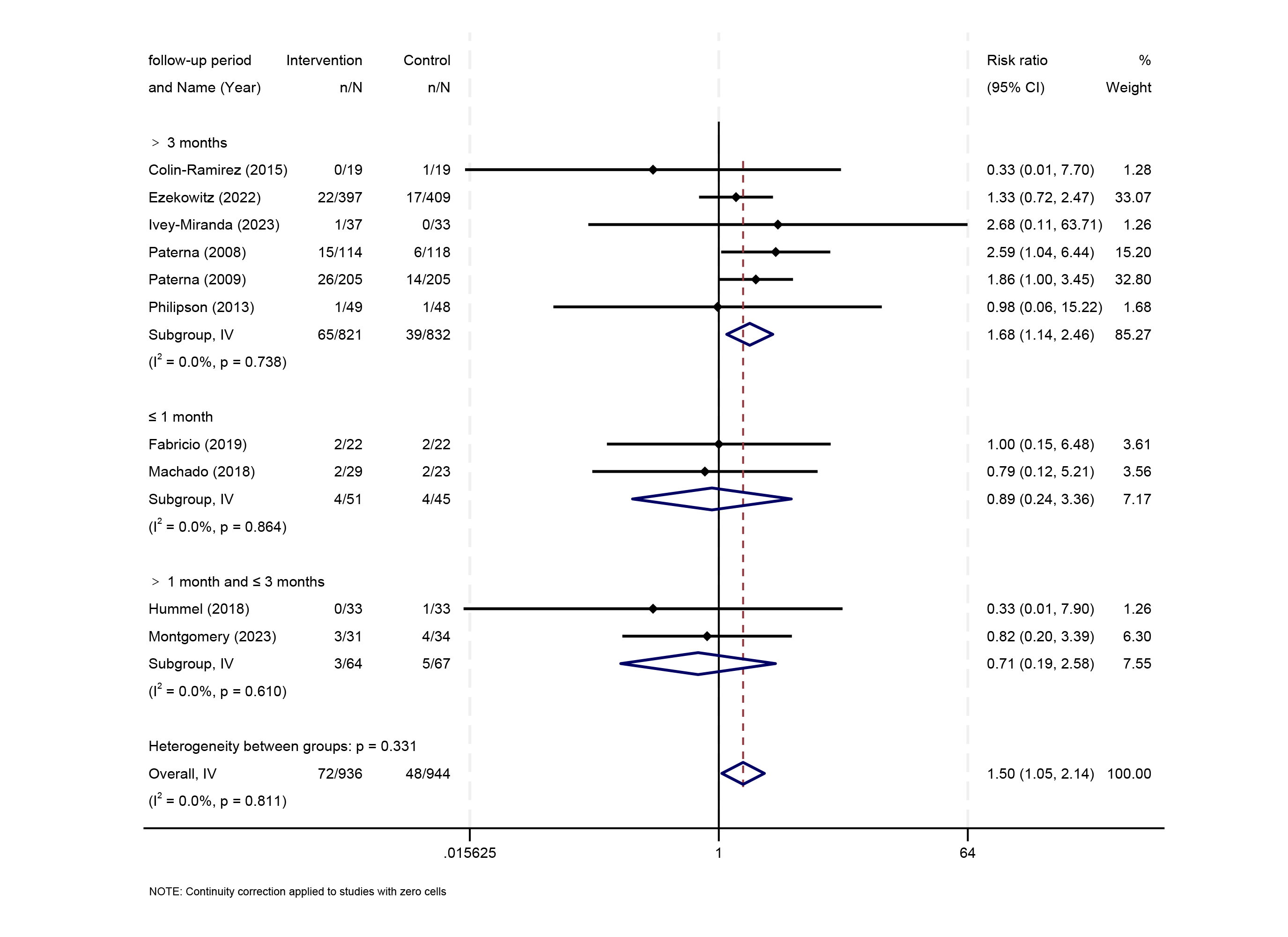

Supplement: Supplementary file 6 [file Image6.tif]

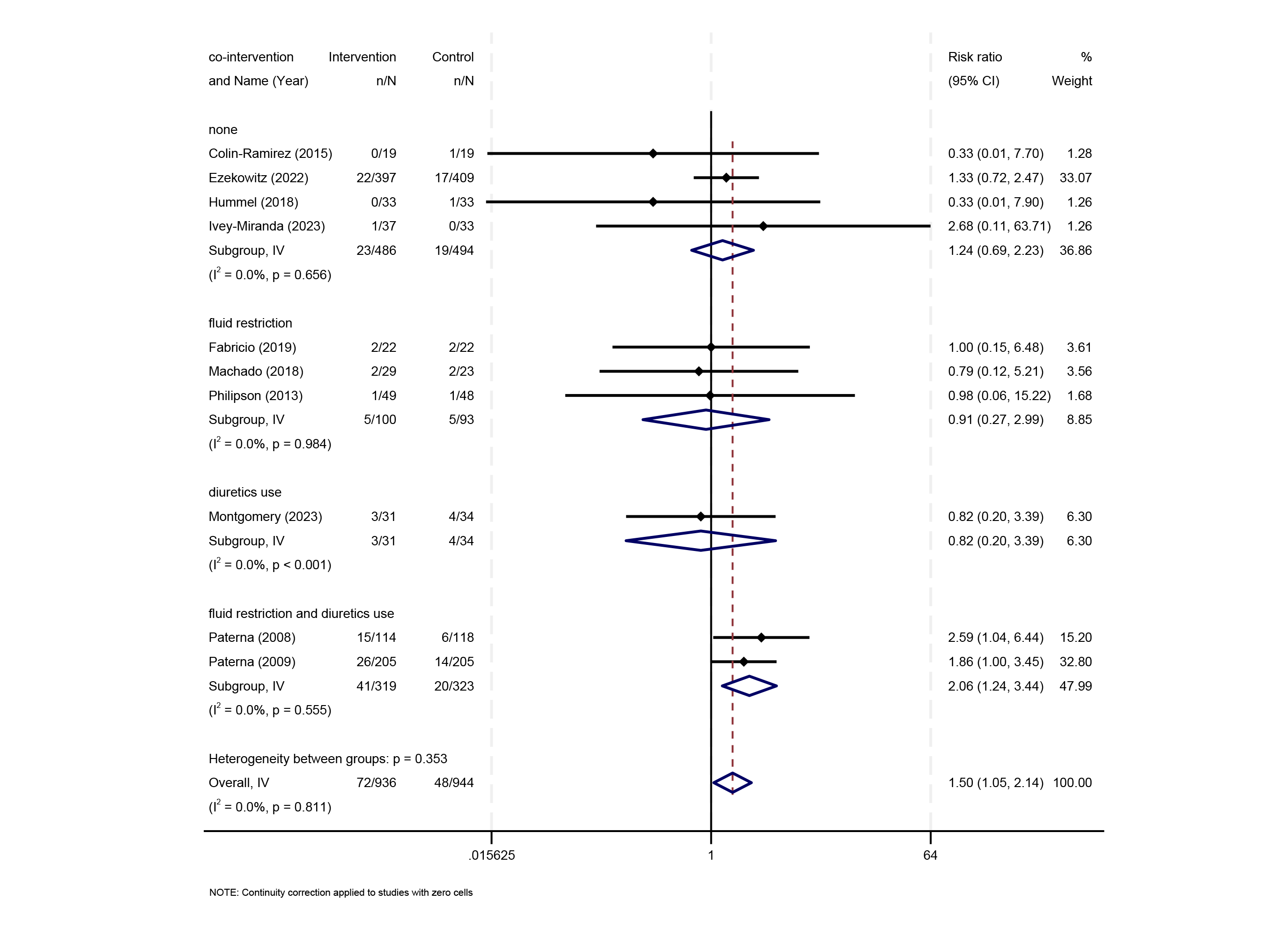

Supplement: Supplementary file 7 [file Image7.tif]

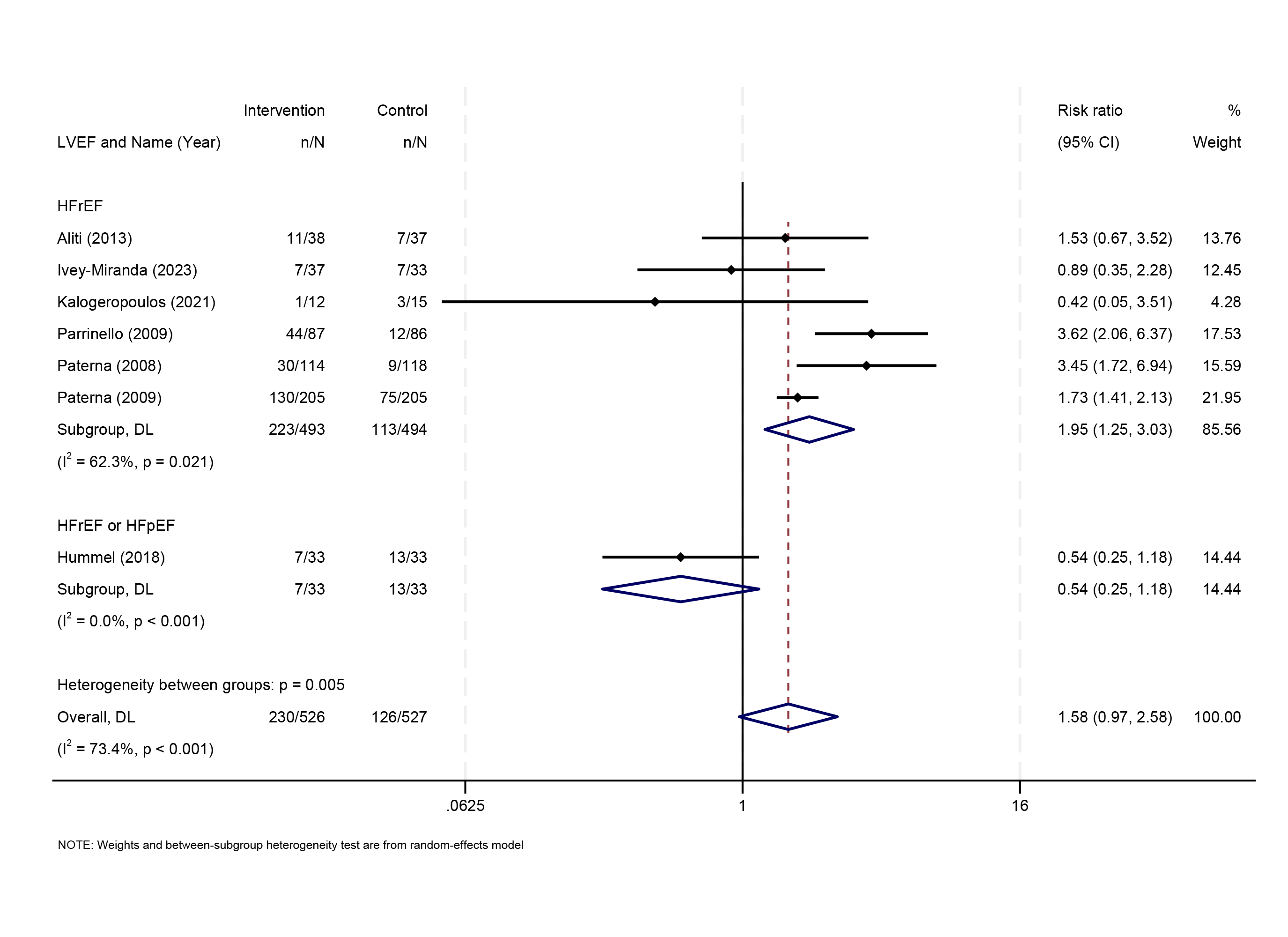

Supplement: Supplementary file 8 [file Image8.tif]

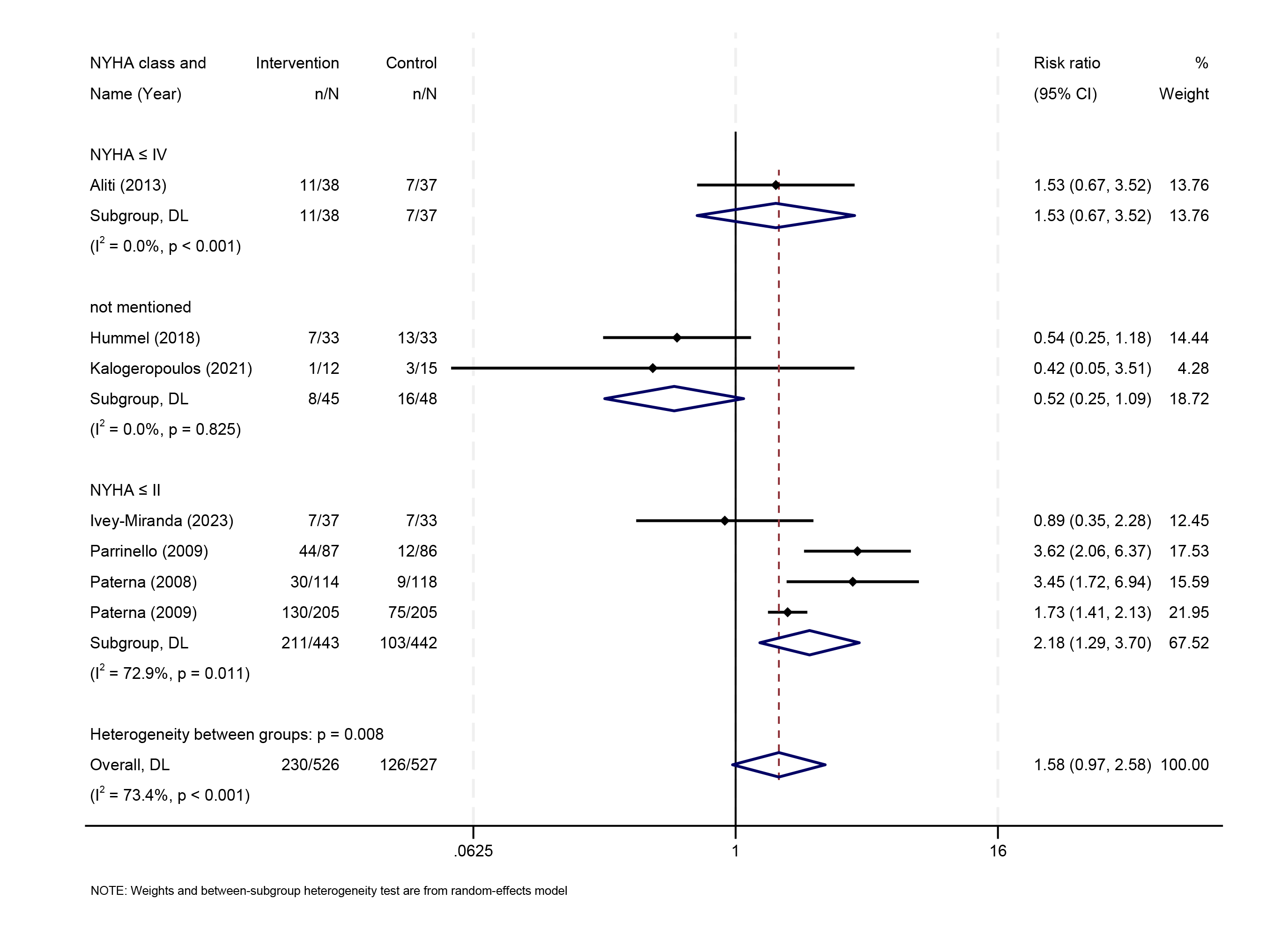

Supplement: Supplementary file 9 [file Image9.tif]

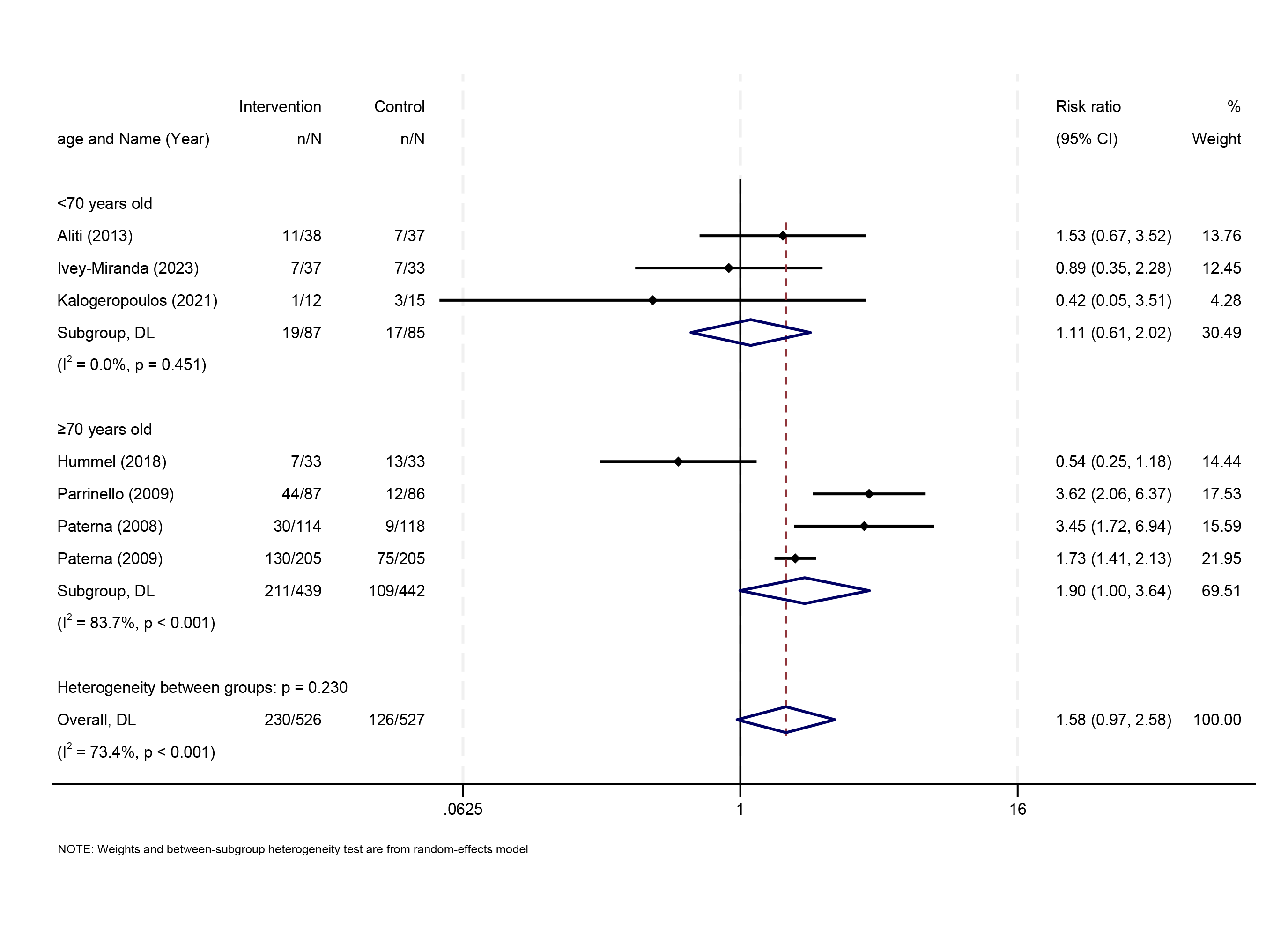

Supplement: Supplementary file 10 [file Image10.tif]

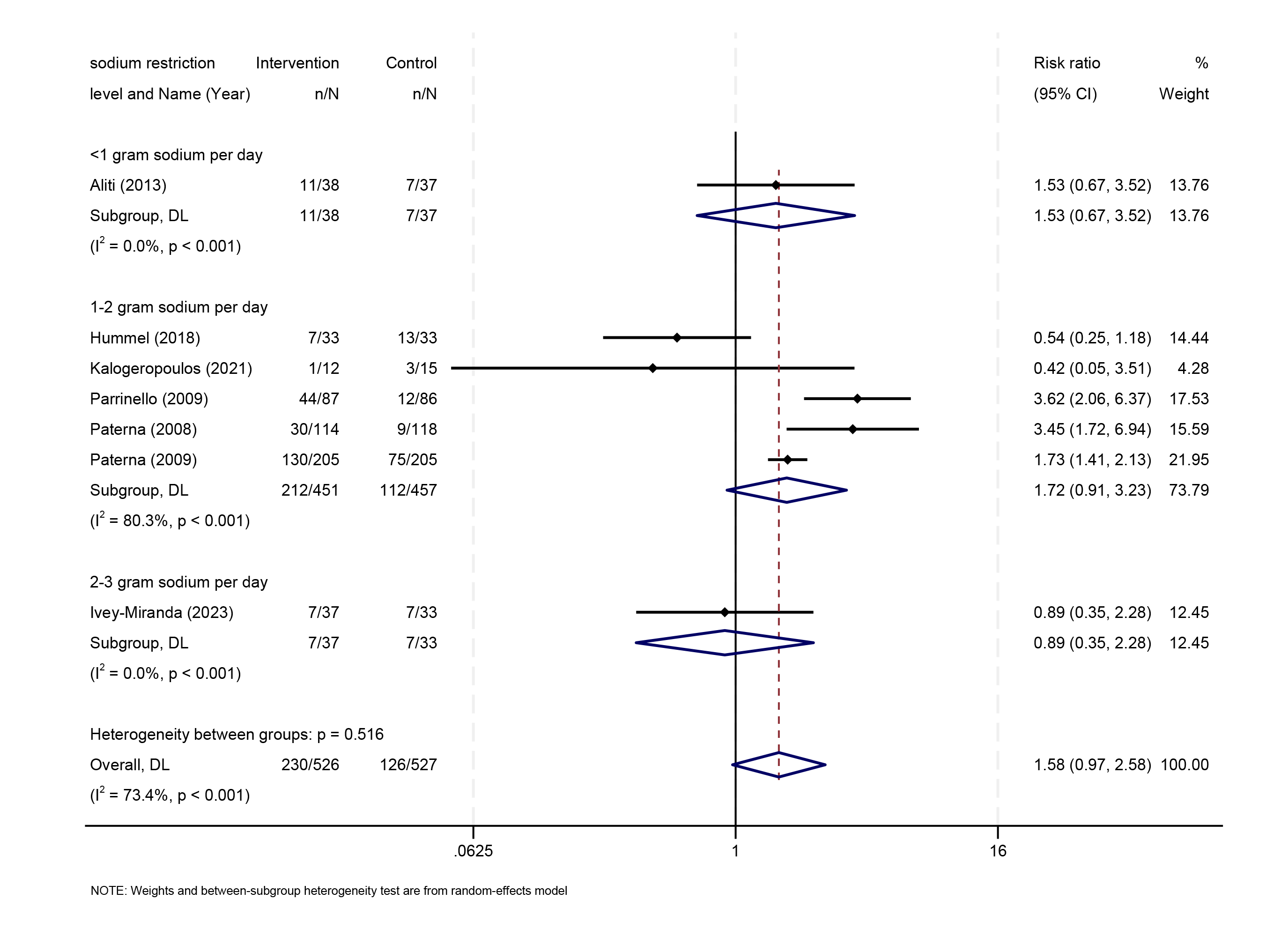

Supplement: Supplementary file 11 [file Image11.tif]

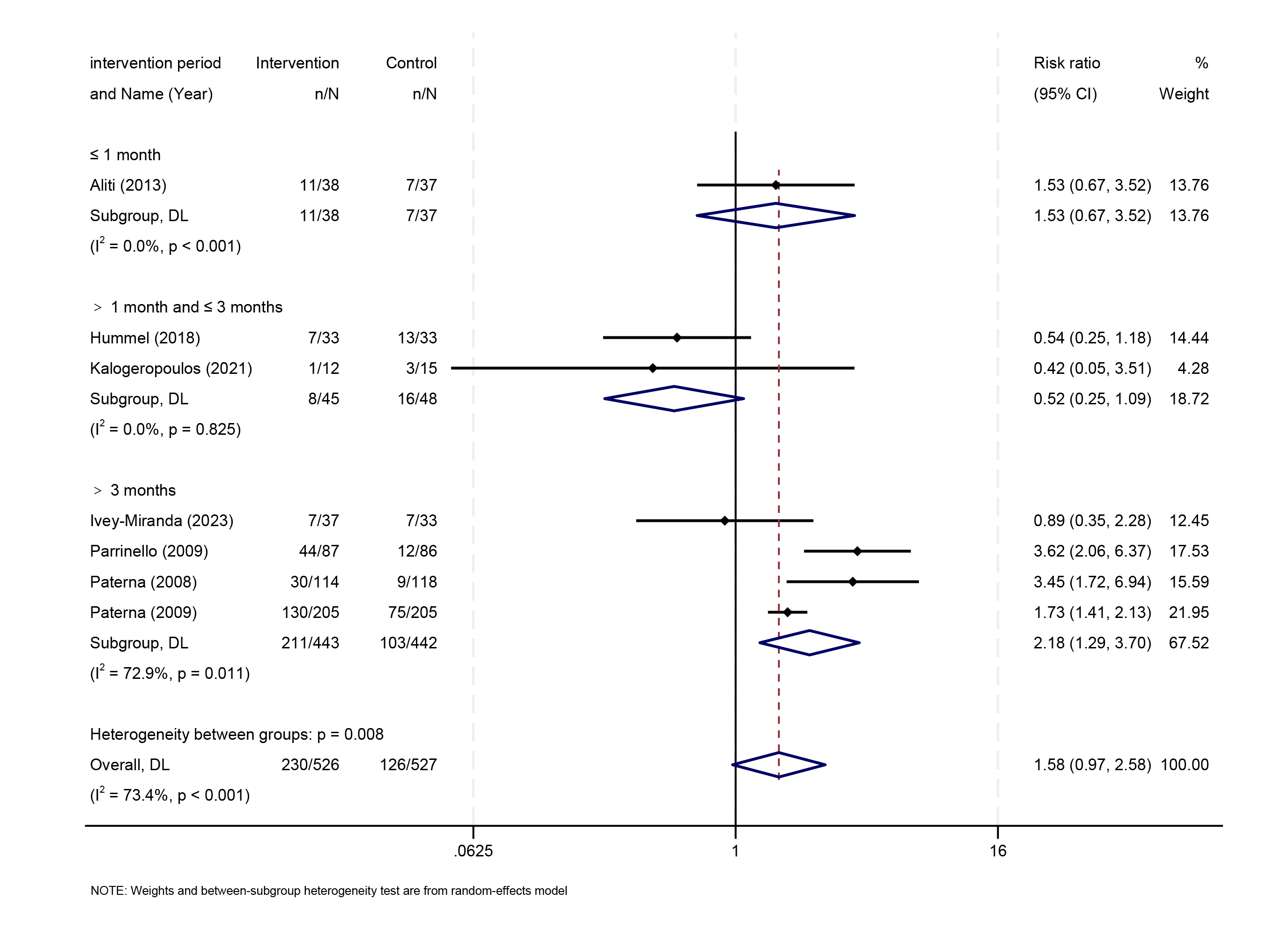

Supplement: Supplementary file 12 [file Image12.tif]

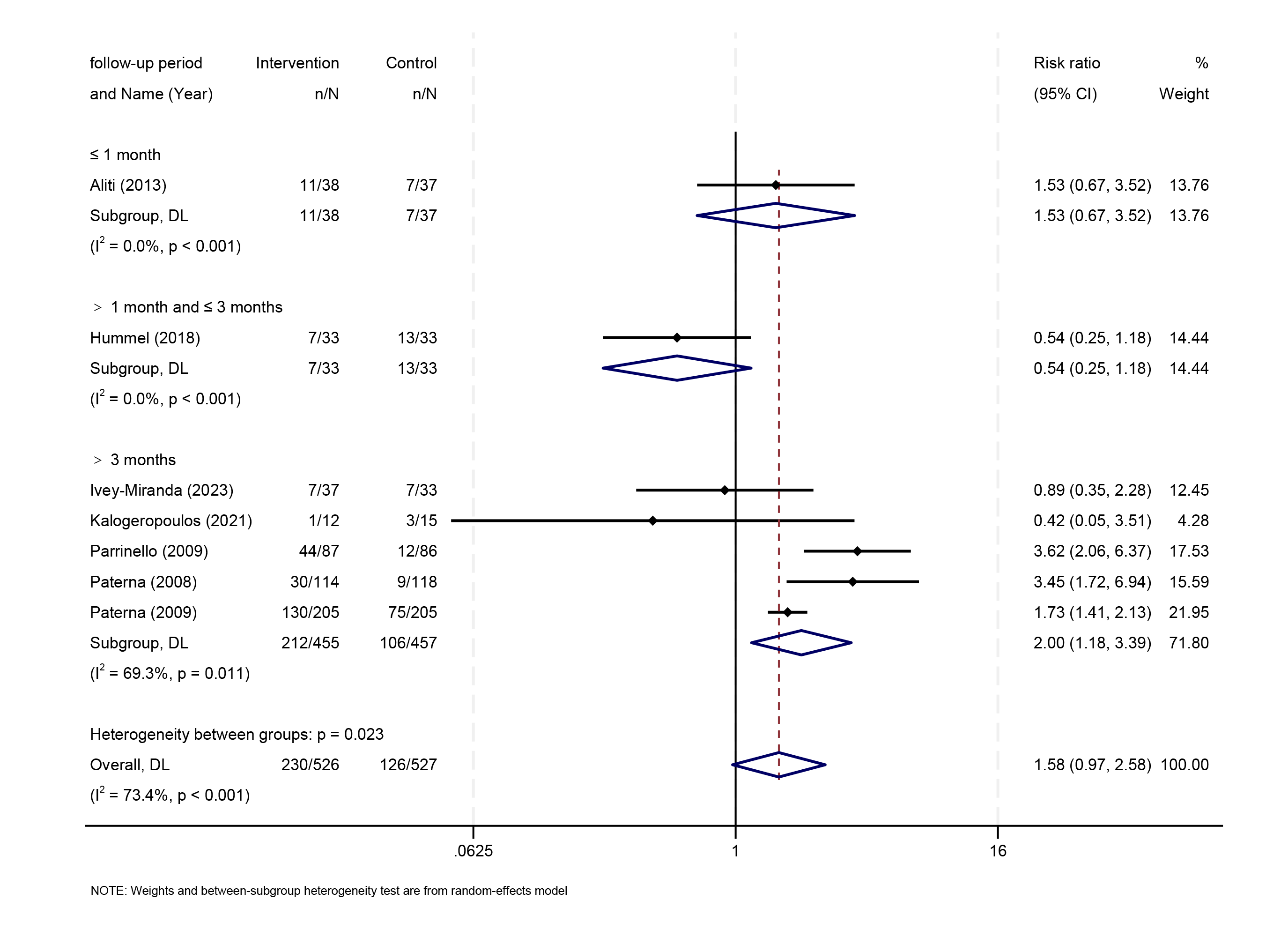

Supplement: Supplementary file 13 [file Image13.tif]

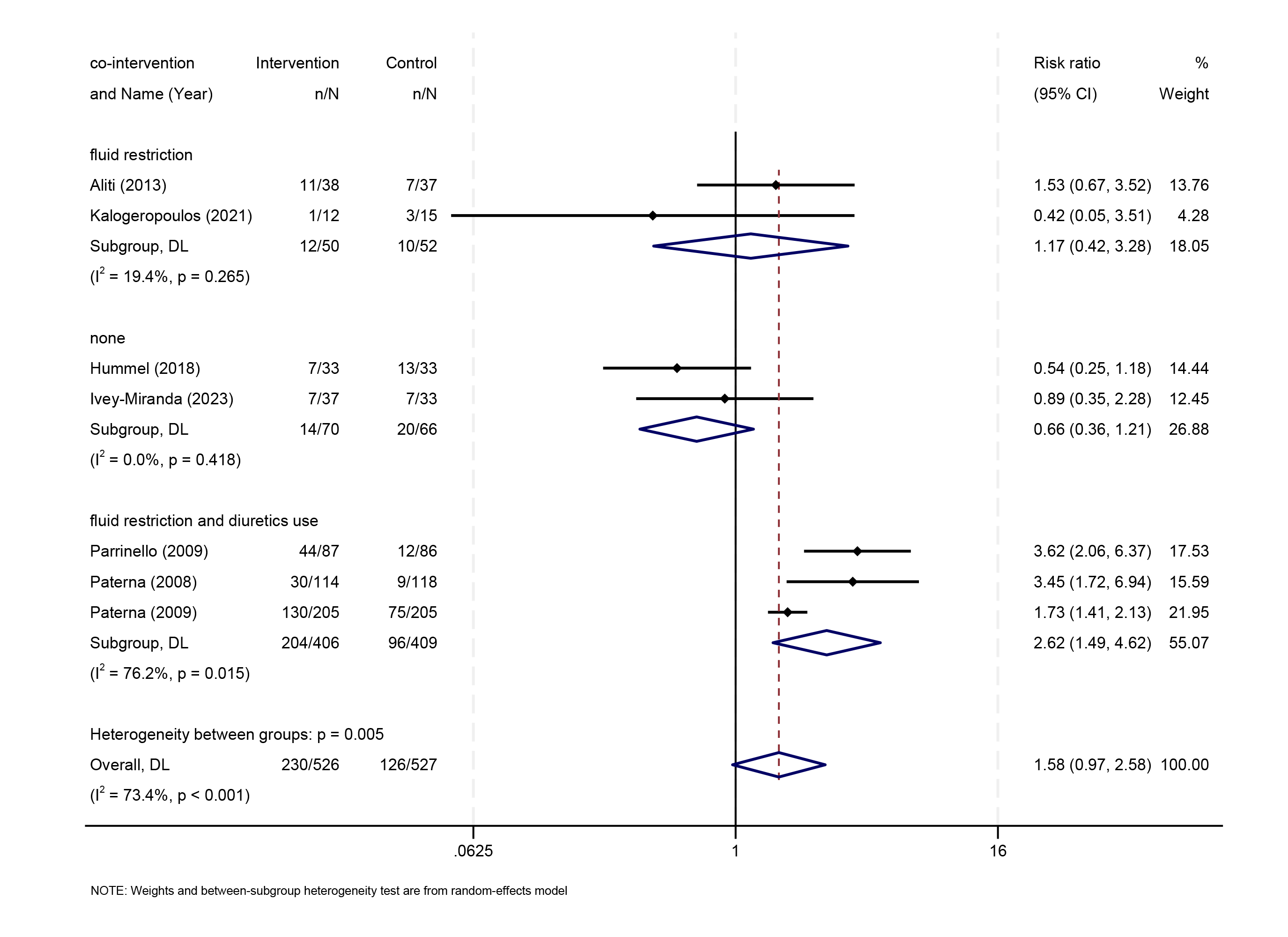

Supplement: Supplementary file 14 [file Image14.tif]

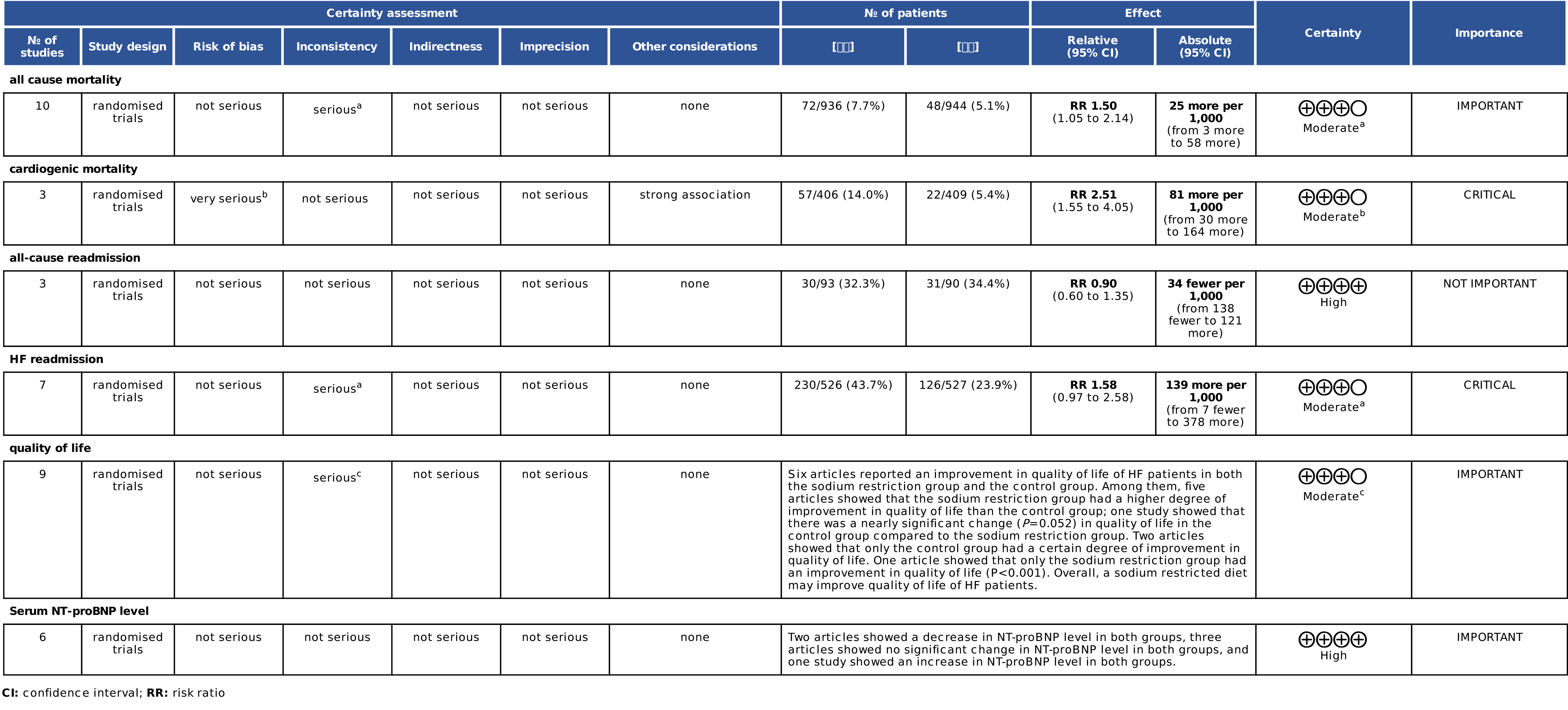

Supplement: Supplementary file 15 [file Image15.tif]
