# Supplementary material for: The effect of sodium restricted diet on the prognosis of heart failure patients: a systemic review and meta-analysis
Source: Front Cardiovasc Med. 2026 May 1;13:1751581. doi: 10.3389/fcvm.2026.1751581 (PMC13176190; doi:10.3389/fcvm.2026.1751581)
Supplement: Supplementary file 16 [file Table1.docx]

Supplementary Table 1 Subgroup analyses summary of all-cause mortality

| **Stratification factor** | **Subgroup** | **Number of included study** | **RR (95%CI)** | **Intragroup heterogeneity** | **Intergroup heterogeneity** |
| --- | --- | --- | --- | --- | --- |
| LVEF | HFrEF or HFpEF | 6 | RR 1.12 [95%CI: 0.67-1.88] | I² = 0.0%, P = 0.895 | P = 0.198 |
|  | HFrEF | 3 | RR 2.08 [95%CI: 1.25-3.44] | I² = 0.0%, P = 0.829 |  |
|  | HFpEF | 1 | RR 0.79 [95%CI: 0.12-5.21] | - |  |
| NYHA class | NYHA ≤ II | 3 | RR 2.08 [95%CI: 1.25-3.44] | I² = 0.0%, P = 0.829 | P = 0.260 |
|  | NYHA ≤ III | 2 | RR 0.61 [95%CI: 0.08-4.85] | I² = 0.0%, P = 0.612 |  |
|  | NYHA ≤ IV | 2 | RR 1.27 [95%CI: 0.70-2.28] | I² = 0.0%, P = 0.607 |  |
|  | not mentioned | 3 | RR 0.79 [95%CI: 0.27-2.29] | I² = 0.0%, P = 0.840 |  |
| mean/median age | <70 years old | 4 | RR 1.27 [95%CI: 0.72-2.24] | I² = 0.0%, P = 0.802 | P = 0.457 |
|  | ≥70 years old | 6 | RR 1.67 [95%CI: 1.06-2.64] | I² = 0.0%, P = 0.593 |  |
| sodium restriction level | 1-2 gram sodium per day | 6 | RR 1.61 [95%CI: 1.10-2.35] | I² = 0.0%, P = 0.583 | P = 0.532 |
|  | 2-3 gram sodium per day | 2 | RR 1.51 [95%CI: 0.19-12.00] | I² = 0.0%, P = 0.637 |  |
|  | <1 gram sodium per day | 2 | RR 0.81 [95%CI: 0.26-2.52] | I² = 0.0%, P = 0.976 |  |
| intervention period | > 3 months | 5 | RR 1.69 [95%CI: 1.15-2.50] | I² = 0.0%, P = 0.626 | P = 0.811 |
|  | ≤ 1 month | 3 | RR 0.86 [95%CI: 0.33-2.26] | I² = 0.0%, P = 0.982 |  |
|  | > 1 month and ≤ 3 months | 2 | RR 0.62 [95%CI: 0.08-4.90] | I² = 0.0%, P = 0.614 |  |
| follow-up period | > 3 months | 6 | RR 1.68 [95%CI: 1.14-2.46] | I² = 0.0%, P = 0.738 | P = 0.331 |
|  | ≤ 1 month | 2 | RR 0.89 [95%CI: 0.24-3.36] | I² = 0.0%, P = 0.864 |  |
|  | > 1 month and ≤ 3 months | 2 | RR 0.71 [95%CI: 0.19-2.58] | I² = 0.0%, P = 0.610 |  |
| co-intervention measures | none | 4 | RR 1.24 [95%CI: 0.69-2.23] | I² = 0.0%, P = 0.656 | P = 0.353 |
|  | fluid restriction | 3 | RR 0.91 [95%CI: 0.27-2.99] | I² = 0.0%, P = 0.984 |  |
|  | diuretics use | 1 | RR 0.82 [95%CI: 0.20-3.39] | - |  |
|  | fluid restriction and diuretics use | 2 | RR 2.06 [95%CI: 1.24-3.44] | I² = 0.0%, P = 0.555 |  |
| Overall | Overall | 10 | RR 1.50 [95%CI: 1.05-2.14] | I² = 0.0%, P = 0.811 | - |

Note: All data are extracted from the subgroup analyses results of all-cause mortality, and continuity correction is applied to studies with zero cells. RR: Risk Ratio; CI: Confidence Interval.
