# Supplementary material for: The effect of sodium restricted diet on the prognosis of heart failure patients: a systemic review and meta-analysis
Source: Front Cardiovasc Med. 2026 May 1;13:1751581. doi: 10.3389/fcvm.2026.1751581 (PMC13176190; doi:10.3389/fcvm.2026.1751581)
Supplement: Supplementary file 17 [file Table2.docx]

Supplementary Table 2 Subgroup analyses summary of HF readmission

| **Stratification factor** | **Subgroup** | **Number of included study** | **RR (95%CI)** | **Intragroup heterogeneity** | **Intergroup heterogeneity** |
| --- | --- | --- | --- | --- | --- |
| LVEF | HFrEF or HFpEF | 1 | RR 0.54 [95%CI: 0.25-1.18] | - | P = 0.005 |
|  | HFrEF | 6 | RR 1.95 [95%CI: 1.25-3.03] | I² = 62.3%, P = 0.021 |  |
| NYHA class | NYHA ≤ IV | 1 | RR 1.53 [95%CI: 0.67-3.52] | - | P = 0.008 |
|  | NYHA ≤ II | 4 | RR 2.18 [95%CI: 1.29-3.70] | I² = 72.9%, P = 0.011 |  |
|  | not mentioned | 2 | RR 0.52 [95%CI: 0.25-1.09] | I² = 0.0%, P = 0.825 |  |
| mean/median age | <70 years old | 3 | RR 1.11 [95%CI: 0.61-2.02] | I² = 0.0%, P = 0.451 | P = 0.230 |
|  | ≥70 years old | 4 | RR 1.90 [95%CI: 1.00-3.64] | I² = 83.7%, P < 0.001 |  |
| sodium restriction level | <1 gram sodium per day | 1 | RR 1.53 [95%CI: 0.67-3.52] | - | P = 0.516 |
|  | 1-2 gram sodium per day | 5 | RR 1.72 [95%CI: 0.91-3.23] | I² = 80.3%, P < 0.001 |  |
|  | 2-3 gram sodium per day | 1 | RR 0.89 [95%CI: 0.35-2.28] | - |  |
| intervention period | ≤ 1 month | 1 | RR 1.53 [95%CI: 0.67-3.52] | - | P = 0.008 |
|  | > 1 month and ≤ 3 months | 2 | RR 0.52 [95%CI: 0.25-1.09] | I² = 0.0%, P = 0.825 |  |
|  | > 3 months | 4 | RR 2.18 [95%CI: 1.29-3.70] | I² = 72.9%, P = 0.011 |  |
| follow-up period | ≤ 1 month | 1 | RR 1.53 [95%CI: 0.67-3.52] | - | P = 0.023 |
|  | > 1 month and ≤ 3 months | 1 | RR 0.54 [95%CI: 0.25-1.18] | - |  |
|  | > 3 months | 5 | RR 2.00 [95%CI: 1.18-3.39] | I² = 69.3%, P = 0.011 |  |
| co-intervention measures | fluid restriction | 2 | RR 1.17 [95%CI: 0.42-3.28] | I² = 19.4%, P = 0.265 | P = 0.005 |
|  | fluid restriction and diuretics use | 3 | RR 2.62 [95%CI: 1.49-4.62] | I² = 76.2%, P = 0.015 |  |
|  | none | 2 | RR 0.66 [95%CI: 0.36-1.21] | I² = 0.0%, P = 0.418 |  |
| Overall | Overall | 7 | RR 1.58 [95%CI: 0.97-2.58] | I² = 73.4%, P < 0.001 | - |

Note: All data are extracted from the subgroup analysis results of HF readmission, and continuity correction is applied to studies with zero cells. RR = Risk Ratio; CI = Confidence Interval. Weights and between-subgroup heterogeneity test are from random-effects model.
