# Supplementary material for: The effect of sodium restricted diet on the prognosis of heart failure patients: a systemic review and meta-analysis
Source: Front Cardiovasc Med. 2026 May 1;13:1751581. doi: 10.3389/fcvm.2026.1751581 (PMC13176190; doi:10.3389/fcvm.2026.1751581)
Supplement: Supplementary file 19 [file Table4.docx]

Table 1 Characteristics of included articles

| **First Author** | **Country** | **Year** | **Types of heart failure** | **NYHA class** | **Renal function** | **Age ( years old )** | **Sample Size ( n )** |
| --- | --- | --- | --- | --- | --- | --- | --- |
|  |  |  |  |  |  | **Sodium restriction group / Control group** | **Sodium restriction group / Control group** |
| Aliti | Brazil | 2013 | Acute decompensated HFrEF | II / III / IV | Estimated creatinine clearance > 30 mL/min/1.73 m^2^ | 60.6 ± 10.5 / 59.3 ± 12.2 | 38 / 37 |
| Colin-Ramirez | Canada | 2004 | HFrEF or HFpEF | I / II / III | Patients with renal failure were excluded | 64.2 ± 16.2 / 59.9 ± 16.4 | 30 / 35 |
| Colin-Ramirez | Canada | 2015 | Chronic HFrEF or HFpEF | II / III | eGFR > 20 mL/min/1.73 m^2^ | 66.1 ( 58.0 - 71.4 ) / 63.9 ( 51.6 - 76.9 ) | 19 / 19 |
| Ezekowitz | Australia, etc | 2022 | Chronic HFrEF or HFpEF | I / II / III / IV | eGFR > 20 mL/min/1.73 m^2^ | 66 ( 57 - 73 ) / 67 ( 58 - 75 ) | 397 / 409 |
| Fabricio | Brazil | 2019 | Acute decompensated HFrEF or HFpEF | - | Estimated creatinine clearance > 30 mL/min/1.73 m^2^ | 59.5 ± 11.9 / 56.4 ± 12.3 | 22 / 22 |
| Hummel | USA | 2018 | Acute decompensated HFrEF or HFpEF | - | Estimated creatinine clearance > 30 mL/min/1.73 m^2^ | 71 ± 8 / 70 ± 8 | 33 / 33 |
| Ivey-Miranda | Mexico | 2023 | Chronic HFrEF | I / II | Estimated creatinine clearance > 30 mL/min/1.73 m^2^ | 61 ± 12 / 58 ± 13 | 37 / 33 |
| Kalogeropoulos | USA | 2019 | HFrEF | - | Patients with renal replacement therapy or Stage 4 or 5 CKD were excluded | 59.5 ± 9.1 / 63.9 ± 11.4 | 12 / 15 |
| Machado d’Almeida | Brazil | 2018 | Decompensated HFpEF | II / III / IV | eGFR > 30 mL/min/1.73 m^2^ | 73.7 ± 11.1 / 70.4 ± 12.6 | 30 / 23 |
| Montgomery | USA | 2023 | Acute HFrEF or HFpEF | - | eGFR > 15 mL/min/1.73 m^2^ | 70 ± 13 / 70 ± 12 | 31 / 34 |
| Nakasato | Brazil | 2010 | Compensated HFrEF | I / II / III | Creatinine < 2.5 mg/dL | 52 ± 2 / 52 ± 2 | 25 / 25 |
| Parrinello | Italy | 2009 | Compensated congestive HFrEF | II | Creatinine < 2 mg/dL, BUN < 60mg/dL | 72.5 ± 8 / 73.1 ± 7 | 87 / 86 |
| Paterna | Italy | 2008 | Compensated congestive HFrEF | II | Creatinine < 2 mg/dL, BUN < 60 mg/dL | 73.3 ± 9 / 72.1 ± 7 | 114 / 118 |
| Paterna | Italy | 2009 | Compensated HFrEF | II | Creatinine < 2 mg/dL, BUN < 60 mg/dL | - | 205 / 205 |
| Philipson | Sweden | 2010 | Chronic HFrEF or HFpEF | II / III | Patients with renal dysfunction ( creatinine > 250 μmol/L ) were excluded | 74 ± 8 / 74 ± 9 | 17 / 13 |
| Philipson | Sweden | 2013 | Chronic HFrEF or HFpEF | II / III | Patients with renal dysfunction ( creatinine > 250 μmol/L ) were excluded | 74 ± 8.6 / 76 ± 7.5 | 49 / 48 |

Note: Age is shown as mean ± standard deviation, or median (quartile 1-quartile 3). HFrEF: heart failure with reduced ejection fraction; HFpEF: heart failure with preserved ejection fraction; NYHA: New York Heart Association; eGFR: estimated glomerular filtration rate; BUN: blood urea nitrogen; CKD: chronic kidney disease
