# Supplementary material for: The effect of sodium restricted diet on the prognosis of heart failure patients: a systemic review and meta-analysis
Source: Front Cardiovasc Med. 2026 May 1;13:1751581. doi: 10.3389/fcvm.2026.1751581 (PMC13176190; doi:10.3389/fcvm.2026.1751581)
Supplement: Supplementary file 20 [file Table5.docx]

Table 2 Intervention details of included articles

| **First Author** | **Country** | **Year** | **Intervention / Follow-up Period** | **Sodium intake of the intervention group** | **Sodium intake of the control group** | **Other Interventions** |
| --- | --- | --- | --- | --- | --- | --- |
|  |  |  |  |  |  |  |
| Aliti | Brazil | 2013 | 7 days / 1 month | 0.8 gram sodium per day | 3 - 5 gram sodium per day | The intervention group's fluid intake was restricted to 800 mL per day |
| Colin-Ramirez | Canada | 2004 | 6 months | 2 - 2.4 gram sodium per day | Follow diet suggestions | The intervention group's fluid intake was restricted to 1.5 L per day |
| Colin-Ramirez | Canada | 2015 | 6 months | 1.5 gram sodium per day | 2.3 gram sodium per day | - |
| Ezekowitz | Australia, etc | 2022 | 12 months / 12 months | 1.5 gram sodium per day | Follow clinical suggestions | - |
| Fabricio | Brazil | 2019 | 7 days | 1.2 gram sodium per day | 2.8 gram sodium per day | Fluid intake was restricted to 1 L per day |
| Hummel | USA | 2018 | 12 weeks | 1.5 gram sodium per day | Follow standardized education | - |
| Ivey-Miranda | Mexico | 2023 | 20 weeks | 2 gram sodium per day | 3 gram sodium per day | - |
| Kalogeropoulos | USA | 2019 | 12 weeks / 12 weeks | 1.5 gram sodium per day | 3 gram sodium per day | Fluid intake was restricted to 2 L per day |
| Machado d’Almeida | Brazil | 2018 | 7 days / 1 month | 0.8 gram sodium per day | 4 gram sodium per day | The intervention group's fluid intake was restricted to 800 mL per day |
| Montgomery | USA | 2023 | 4 days / 3 months | 0.8 gram sodium per day | 0.8 gram sodium per day + 0.8 gram sodium / 2 capsules, tid | Intravenous injection of furosemide > 10 mg per hour |
| Nakasato | Brazil | 2010 | 7 days | 0.8 gram sodium per day | 2.4 gram sodium per day | Fluid intake was restricted to 1 L per day |
| Parrinello | Italy | 2009 | 6 months | 1.8 gram sodium per day | 2.8 gram sodium per day | Oral administration of 250 - 500 mg furosemide, bid, fluid intake was restricted to 1 L per day |
| Paterna | Italy | 2008 | 6 months | 1.8 gram sodium per day | 2.8 gram sodium per day | Oral administration of 250 - 500 mg furosemide, bid, fluid intake was restricted to 1 L per day |
| Paterna | Italy | 2009 | 6 months | 1.8 gram sodium per day | 2.8 gram sodium per day | Oral administration of 250 - 500 mg furosemide, bid, fluid intake was restricted to 1 - 2 L per day |
| Philipson | Sweden | 2010 | 12 weeks | 2 - 3 gram sodium per day | Follow ESC guidelines | The intervention group's fluid intake was restricted to 1.5 L per day |
| Philipson | Sweden | 2013 | 12 weeks / 12 months | 2 - 3 gram sodium per day | Follow clinical suggestions | The intervention group's fluid intake was restricted to 1.5 L per day |

ESC: European Society of Cardiology
